# *Rhodiola rosea*, *Ginkgo biloba*, and Ashwagandha as novel antidepressant supplements: converging monoaminergic, neurotrophic, anti-inflammatory, and brain health pathways in depressive disorders

**DOI:** 10.3389/fnut.2026.1762061

**Published:** 2026-03-12

**Authors:** Xikun Li, Xuyi Li, Yunchu Li, Zhishuo Liu, Yihan Cai, Qinqin Yao, Shuang Zhao, Qinming Yu

**Affiliations:** Heilongjiang University of Chinese Medicine, School of Humanities and Management, Harbin, Heilongjiang, China

**Keywords:** antidepressant effects, Ashwagandha, depressive disorder, Rhodiola, supplements

## Abstract

Depressive disorder represents a multifaceted and intricate condition characterized by disturbances in monoaminergic signaling, neurotrophic support mechanisms, and the regulation of inflammatory processes. An increasing body of evidence indicates that natural bioactive compounds may provide adjunctive therapeutic advantages with a reduced incidence of adverse effects in comparison to traditional antidepressants. This review investigates the antidepressant efficacy of *Rhodiola rosea*, *Ginkgo biloba*, and Ashwagandha, three notable herbal supplements endowed with a variety of neurobiological actions. *Rhodiola rosea* is known to elevate monoamine concentrations and modulate pathways involved in the stress response, whereas *Ginkgo biloba* is recognized for its ability to enhance cerebral perfusion, safeguard against oxidative damage, and facilitate synaptic plasticity. Ashwagandha is distinguished by its robust adaptogenic and anti-inflammatory properties, its capacity to diminish cortisol levels, and its role in promoting neurotrophic factors such as brain-derived neurotrophic factor. Collectively, these botanical agents target critical pathways associated with depression, encompassing serotonergic, dopaminergic, and noradrenergic regulation, neurogenesis, mitochondrial functionality, and immunomodulatory processes. Preclinical studies and emerging clinical evidence suggest their potential in alleviating depressive symptoms, fostering resilience, and augmenting overall mental health. This review integrates mechanistic insights and translational findings, highlighting the promise of these natural compounds as adjunctive strategies in depression management.

## 1 Introduction

Depression constitutes one of the most incapacitating neuropsychiatric disorders globally (1). Despite considerable progress in the field of psychopharmacology, traditional antidepressant therapies persistently exhibit significant shortcomings, such as delayed therapeutic onset, suboptimal rates of remission, elevated instances of partial response, and adverse side-effect profiles (2, 3). These limitations have catalyzed an increased global focus on nutraceuticals and phytomedicines possessing multimodal biological activities that transcend mere monoamine reuptake inhibition. Notably, *Rhodiola rosea*, *Ginkgo biloba*, and *Withania somnifera* (Ashwagandha) have garnered particular interest owing to their distinctive pharmacological characteristics that intersect with monoaminergic neurotransmission, neurotrophic support, modulation of the hypothalamic-pituitary-adrenal (HPA) axis, and anti-inflammatory signaling—mechanisms that are consistently implicated in the pathophysiological framework of depression. Although numerous scholarly articles have assessed the general antidepressant characteristics of specific botanical entities, there exists a significant deficiency of a thorough and mechanistically cohesive evaluation that amalgamates the converging molecular pathways of Rhodiola, Ginkgo, and Ashwagandha within the monoaminergic, neurotrophic, and immunomodulatory spheres. Current reviews tend to either concentrate on individual herbs devoid of comparative analysis, address broad classifications of adaptogens without meticulous mechanistic triangulation, or highlight clinical outcomes while inadequately correlating them with molecular biology. Consequently, a comprehensive review that rigorously investigates how these three botanicals influence intersecting neurobiological circuits related to depressive disorders, while simultaneously translating these mechanistic revelations into clinical ramifications, is both timely and imperative.

The extant body of scholarly work indicates that these botanical supplements manifest their antidepressant properties not merely through straightforward pharmacodynamic mechanisms but rather via extensive modulation of neurochemical networks at a systemic level. For instance, *Rhodiola rosea* augments serotonergic, dopaminergic, and noradrenergic signaling, in part due to its inhibition of monoamine oxidases and its modulation of presynaptic neurotransmitter transporters (4–6). Ashwagandha affects monoaminergic systems indirectly by normalizing hyperactivity of the HPA axis and modulating GABAergic tone, which consequently enhances the balance of serotonergic and dopaminergic systems (7, 8). Conversely, *Ginkgo biloba* enhances monoaminergic activity through its facilitation of neurovascular coupling and the antioxidant stabilization of synaptic microenvironments, thereby offering a complementary mechanism to traditional monoaminergic antidepressants (9–11). These results underscore that the monoaminergic influences of the three herbal agents function within broader adaptogenic and neuroendocrine contexts rather than as mere isolated modifications of neurotransmitter levels.

Beyond the realm of neurotransmission, an essential facet common to these botanical agents is their influence on neuroplasticity. Major depressive disorder is intricately linked with compromised neurogenesis and diminished expression of neurotrophic factors, particularly brain-derived neurotrophic factor (BDNF). Rhodiola has been demonstrated to enhance BDNF levels and activate the ERK/CREB signaling pathway (12), thereby facilitating neuronal survival and promoting neurogenesis within the hippocampus. Ashwagandha has been observed to stimulate neurite outgrowth, augment synaptic protein expression (13), which collectively endorse both structural and functional neural repair mechanisms. *Ginkgo biloba* plays a significant role in neuroplasticity by safeguarding mitochondrial integrity and mitigating glucocorticoid-induced dendritic atrophy (14). These findings underscore a common neurotrophic profile among the three botanicals, thereby reinforcing the notion that effective antidepressant interventions should aim to restore neural circuitry rather than solely modulate neurotransmitter activity. Furthermore, the dysregulation of inflammatory processes is increasingly acknowledged as a pivotal factor in the etiology of depressive disorders (15). Sustained elevations in interleukin-6 (IL-6), tumor necrosis factor-alpha (TNF-α), and other pro-inflammatory cytokines have been observed in individuals diagnosed with major depressive disorder. Each of the three botanical agents demonstrates anti-inflammatory properties through distinct yet interrelated biological pathways. Rhodiola exhibits a downregulation of nuclear factor kappa B (NF-κB) activity and diminishes systemic cytokine production (16). Ashwagandha attenuates microglial activation and restores peripheral inflammatory markers to normative levels (17). *Ginkgo biloba* mitigates cytokine release induced by oxidative stress while maintaining the integrity of endothelial and neuronal cells (18, 19). These findings underscore a significant therapeutic perspective: by implementing substantial immunomodulatory effects, these botanical agents engage biological mechanisms that are not comprehensively addressed by traditional antidepressant treatments.

Notwithstanding the promising findings, the existing clinical literature exhibits significant inconsistencies. While numerous randomized clinical trials substantiate the antidepressant efficacy of Rhodiola, Ginkgo, and Ashwagandha, these investigations are frequently hindered by limited sample sizes, diverse dosing protocols, variability in extract standardization, and insufficient integration of mechanistic biomarkers. Furthermore, a paucity of studies has directly contrasted these botanical agents or explored their potential synergistic effects, thereby leaving crucial inquiries regarding therapeutic optimization unresolved. These deficiencies underscore the necessity for integrative assessments that connect molecular mechanisms with clinical results. Integrating the available body of evidence suggests that depression, understood as a complex disorder, may derive significant advantages from therapeutic interventions that can concurrently rectify neurotransmitter dysregulation, enhance neuroplastic function, and mitigate chronic inflammatory processes. *Rhodiola rosea*, *Ginkgo biloba*, and Ashwagandha demonstrate precisely such multifaceted therapeutic profiles and correspond with contemporary interpretations of depression as a condition characterized by diminished neurobiological resilience. Their commendable safety profiles and compatibility with current antidepressant modalities further emphasize their potential contributions to personalized, integrative mental health care. Nevertheless, the implementation of these findings into standard clinical practice will necessitate more rigorous, standardized, and mechanistically informed clinical investigations. Looking forward, subsequent research endeavors must emphasize biomarker-driven clinical trials that amalgamate neurotrophin concentrations, inflammatory biomarkers, and neurotransmitter metabolites to clarify mechanistic pathways and delineate patient cohorts that are most likely to derive therapeutic benefits from each botanical intervention. The incorporation of genomic, microbiome, and neuroendocrine profiles may further refine individualized treatment strategies. Comparative investigations and combinatorial trials will be imperative to ascertain the presence of synergistic interactions among the three botanicals, as their underlying mechanisms imply complementary rather than superfluous functions. Enhanced phytochemical characterization and pharmacokinetic analysis will be vital for the standardization of extracts and for gaining regulatory endorsement. Ultimately, longitudinal safety assessments and relapse-prevention trials are essential to comprehensively validate the therapeutic efficacy of these botanicals in the context of chronic depressive disorders.

The selection of *Rhodiola rosea*, *Ginkgo biloba*, and *Withania somnifera* (Ashwagandha) in this review is intentional and mechanistically grounded rather than arbitrary. These three botanical agents were chosen because they represent distinct yet complementary phytotherapeutic paradigms that converge on the principal neurobiological processes underlying depressive disorders. While *Rhodiola rosea* primarily enhances stress resilience and monoaminergic signaling through adaptogenic modulation of the hypothalamic-pituitary-adrenal axis (20), *Ginkgo biloba* exerts prominent neurovascular, antioxidant, and mitochondrial-protective effects that support cognitive and synaptic integrity (21). In contrast, Ashwagandha robustly targets neuroendocrine imbalance and neuroinflammation while promoting neurotrophic signaling and cortisol normalization (22). Together, these agents collectively influence serotonergic, dopaminergic, and noradrenergic transmission, brain-derived neurotrophic factor–mediated plasticity, inflammatory cascades, oxidative stress, and mitochondrial bioenergetics, pathways that are increasingly recognized as interdependent in the pathophysiology of depression (23). Moreover, these botanicals originate from diverse traditional medical systems, traditional Chinese medicine, European herbal medicine, and Ayurveda, yet converge on shared molecular targets, providing a unique opportunity to examine depression through a cross-cultural, systems-biology lens. Unlike prior reviews that focus on individual herbs or broadly defined adaptogens, the present review integrates mechanistic, preclinical, and clinical evidence across these three agents to highlight their complementary rather than redundant actions. This integrative framework may offer valuable insights into rational adjunctive use, biomarker-driven stratification, and the development of multi-target therapeutic strategies for depressive disorders.

## 2 Methodology

A comprehensive and structured literature search was conducted to identify preclinical, translational, and clinical evidence related to the antidepressant effects and underlying neurobiological mechanisms of *Rhodiola rosea*, *Ginkgo biloba*, and *Withania somnifera* (Ashwagandha). The search was performed across multiple electronic databases, including PubMed/MEDLINE, Web of Science, Scopus, and Google Scholar, to ensure broad coverage of biomedical, pharmacological, and neuroscientific literature. Articles published up to March 2025 were considered eligible for inclusion.

Search strategies combined the scientific and common names of each botanical with depression-related and mechanistic keywords. Representative search terms included: “*Rhodiola rosea*” *OR* “*Rhodiola*,” “*Ginkgo biloba*,” “*Withania somnifera*” *OR* “*Ashwagandha*” in combination with “*depression*,” “*antidepressant*,” “*monoaminergic neurotransmission*,” “*BDNF*,” “*neuroplasticity*,” “*HPA axis*,” “*neuroinflammation*,” “*oxidative stress*,” “*mitochondrial dysfunction*,” and “*clinical trial*.” Boolean operators and database-specific filters were applied where appropriate to optimize sensitivity and specificity.

Eligible publications included peer-reviewed original research articles, encompassing *in vivo* and *in vitro* experimental studies, randomized controlled trials, observational clinical studies, and mechanistically relevant translational investigations. High-quality review articles and meta-analyses were also consulted to contextualize findings and identify additional primary literature. Studies were restricted to those published in English. Exclusion criteria comprised studies lacking relevance to depressive disorders, investigations focused solely on unrelated neurological or systemic conditions, reports involving non-standardized or poorly characterized herbal preparations, and articles without sufficient methodological or outcome detail.

To enhance comprehensiveness, manual screening of reference lists from key articles and recent reviews was conducted to identify additional relevant studies not captured through database searches. When multiple publications addressed similar experimental models or clinical populations, priority was given to studies with larger sample sizes, standardized extracts, rigorous study designs, and clearly reported outcomes.

Given the objective of this article as a narrative and mechanistic integrative review, formal quantitative synthesis or meta-analysis was not undertaken. Instead, the selected literature was analyzed and synthesized thematically, with studies grouped according to biological pathways (e.g., monoaminergic modulation, neurotrophic signaling, HPA-axis regulation, neuroinflammation, oxidative stress, and mitochondrial function) and level of evidence (preclinical versus clinical). Particular emphasis was placed on studies that linked molecular and cellular mechanisms with behavioral or clinical outcomes, thereby enhancing translational relevance. Dosages are reported as mg/day of extract where available, with key phytochemical standardization noted when specified in the original studies.

Collectively, this approach was designed to provide a comprehensive, balanced, and mechanistically coherent synthesis of the existing evidence, while acknowledging variability in study quality, extract standardization, and clinical methodology across the literature.

## 3 Pathophysiology of depression

### 3.1 Monoaminergic dysfunction

Monoaminergic disruptions especially those related to serotonin (5-HT), norepinephrine (NE), and dopamine (DA), constitute a fundamental and classical mechanism in the pathophysiological framework of depression. Diminished levels of monoamines, altered sensitivity of receptors, and dysregulated transport mechanisms such as SERT and NET play a significant role in the manifestation of depressive symptoms (24). Neuroimaging investigations reveal a reduction in 5-HT and NE release, alongside compromised mesolimbic dopaminergic reward pathways in individuals diagnosed with depression (25). Prolonged exposure to stress further elevates the activity of monoamine oxidase-A (MAO-A) while concurrently diminishing monoamine biosynthesis, thereby exacerbating depressive-like manifestations (26).

### 3.2 HPA-axis dysregulation

Dysregulation of the hypothalamic-pituitary-adrenal (HPA) axis constitutes a significant characteristic of major depressive disorder. Prolonged exposure to stress results in the increased secretion of corticotropin-releasing hormone (CRH) and adrenocorticotropic hormone (ACTH), culminating in hypercortisolemia (27). Sustained elevation of cortisol levels leads to atrophy of the hippocampus, hinders neurogenesis, and disturbs cognitive and emotional functions (28). In clinical practice, a notable 40–60% of individuals diagnosed with depression demonstrate nonsuppression in the dexamethasone suppression test, indicative of compromised negative feedback mechanisms within the HPA axis (29).

### 3.3 Neuroinflammation

Neuroinflammation has emerged as a central and biologically plausible contributor to the pathophysiology of depressive disorders, bridging immune dysregulation with alterations in neurotransmission, neuroplasticity, and stress responsivity (30, 31). Clinical studies consistently report elevated circulating and central levels of pro-inflammatory cytokines, including interleukin-1β (IL-1β), interleukin-6 (IL-6), and tumor necrosis factor-α (TNF-α), in patients with major depressive disorder, with cytokine concentrations often correlating with symptom severity, illness chronicity, and treatment resistance (30, 32). Peripheral inflammatory signals can access the central nervous system through humoral, neural, and cellular pathways, including cytokine transport across the blood–brain barrier and vagal nerve signaling, thereby initiating or amplifying central immune activation (31).

Within the brain, microglia and astrocytes represent the primary cellular mediators of neuroinflammatory responses. Chronic stress and inflammatory stimuli promote microglial activation, leading to sustained release of pro-inflammatory cytokines, reactive oxygen and nitrogen species, and excitotoxic mediators that disrupt synaptic function and neuronal integrity (30). Activated glial cells interfere with monoaminergic neurotransmission by reducing serotonin synthesis, altering dopamine signaling, and impairing norepinephrine regulation, thereby directly contributing to mood dysregulation and anhedonia (31). A critical mechanistic link between inflammation and depression involves activation of the indoleamine-2,3-dioxygenase (IDO) enzyme, which diverts tryptophan metabolism away from serotonin production toward the kynurenine pathway. This metabolic shift reduces central serotonin availability while increasing neuroactive kynurenine metabolites, such as quinolinic acid, which enhance glutamatergic neurotransmission and promote excitotoxicity. These processes impair hippocampal neurogenesis and synaptic plasticity, both of which are essential for emotional regulation and stress adaptation (32, 33).

Neuroinflammation also interacts bidirectionally with dysregulation of the hypothalamic–pituitary–adrenal (HPA) axis. Pro-inflammatory cytokines stimulate corticotropin-releasing hormone (CRH) secretion and impair glucocorticoid receptor signaling, resulting in diminished negative feedback and sustained hypercortisolemia. Elevated cortisol levels, in turn, exacerbate microglial activation and inflammatory signaling, establishing a self-perpetuating cycle of stress, immune activation, and neuronal dysfunction (32, 33). Importantly, neuroinflammatory processes are closely intertwined with oxidative stress and mitochondrial dysfunction, as inflammatory mediators increase reactive oxygen species production and compromise mitochondrial bioenergetics, further amplifying neuronal vulnerability (33). Collectively, these findings support the view that neuroinflammation is not merely an epiphenomenon of depression but a driving force that integrates immune activation with neurotransmitter imbalance, impaired neuroplasticity, and stress-related neuroendocrine dysregulation (30, 31). Targeting neuroinflammatory pathways therefore represents a critical therapeutic avenue for the development of novel and adjunctive antidepressant strategies (32, 34).

### 3.4 Oxidative stress

Oxidative stress has been increasingly recognized as a significant biological factor associated with depressive disorders, characterized by an imbalance between the production of reactive oxygen species (ROS) and the body’s antioxidant defense mechanisms, leading to cellular damage and dysregulated neuronal function (35–37). Major depressive disorder (MDD) and related depressive conditions exhibit elevated oxidative stress markers, reflecting increased oxidative damage to lipids, proteins, and DNA, as well as a relative depletion of antioxidants in plasma and peripheral tissues (35–37). The brain is particularly susceptible to oxidative stress due to its high oxygen consumption, rich lipid content, and comparatively limited antioxidant capacity, making redox imbalance a salient contributor to neuronal dysfunction within depressive pathophysiology (35–37).

Elevated levels of reactive oxygen species can activate pro-inflammatory signaling cascades and apoptotic pathways, which in turn exacerbate neuroinflammation, impair neurogenesis, and contribute to synaptic and structural changes implicated in depression (104). In a systematic review and meta-analysis, depression was associated with significantly increased oxidative stress as measured by biomarkers of oxidative DNA damage (8-OHdG) and lipid peroxidation (F₂-isoprostanes) compared to non-depressed controls (35–37). The meta-analytic evidence demonstrated that both 8-OHdG and F₂-isoprostanes were elevated in individuals with depression, indicating enhanced oxidative damage, with effect sizes suggesting robust associations across multiple studies (35–37). Although measures of oxidative stress vary by methodology and sample characteristics, the overall pattern of increased oxidative stress in depressive disorders supports a model in which redox imbalance is integrally linked to the neurobiology of depression (35–37). Together, these findings provide compelling evidence that oxidative stress plays a critical role in the pathophysiology of depression and may represent both a biomarker of disease severity and a potential target for therapeutic intervention through modulation of antioxidant defenses (35–37).

### 3.5 Neurotrophic deficits and impaired hippocampal neurogenesis

The diminishment of neurotrophic signaling, especially that related to brain-derived neurotrophic factor (BDNF), stands as a defining characteristic of depression. Diminished BDNF concentrations within the realms of the hippocampus and prefrontal cortex result in hindered synaptogenesis, diminished neuroplasticity, and a slowdown in neurogenesis (38). The BDNF–TrkB pathway plays a crucial role in fostering neuronal resilience, and studies have indicated that antidepressant therapies can elevate BDNF production (39). Prolonged stress curtails BDNF and CREB expression through glucocorticoid-driven mechanisms, amplifying susceptibility to depressive conditions (40).

In addition to generalized neurotrophic deficits, impaired adult hippocampal neurogenesis has emerged as a central and independent pathophysiological mechanism in depressive disorders (41). The hippocampus retains the capacity for lifelong neurogenesis within the dentate gyrus, a process that is critically involved in emotional regulation, stress adaptation, and cognitive flexibility (41). Chronic stress, elevated glucocorticoid exposure, and neuroinflammatory signaling suppress the proliferation, differentiation, and survival of neural progenitor cells, leading to reduced hippocampal volume and functional impairments commonly observed in major depressive disorder (42).

Brain-derived neurotrophic factor (BDNF) plays a pivotal role in regulating adult hippocampal neurogenesis through activation of TrkB-mediated CREB signaling pathways. Clinical and postmortem studies consistently demonstrate reduced BDNF expression and diminished neurogenic capacity in individuals with depression, while effective antidepressant treatments, including SSRIs, SNRIs, and electroconvulsive therapy, restore neurogenesis and promote neuronal maturation. These observations support the neurogenic hypothesis of depression, which posits that impaired hippocampal neurogenesis contributes directly to mood dysregulation and treatment resistance (42, 43).

Importantly, hippocampal neurogenesis is highly sensitive to inflammatory cytokines, oxidative stress, and mitochondrial dysfunction, further linking this mechanism to broader neuroimmune and metabolic abnormalities in depression. Consequently, therapeutic strategies that simultaneously enhance neurotrophic signaling and restore neurogenesis may offer superior and more durable antidepressant effects compared with monoaminergic modulation alone (44, 45).

### 3.6 Mitochondrial dysfunction

Mitochondrial irregularities unveil themselves as a burgeoning and pivotal force driving the depths of major depression. Individuals often display diminished ATP production, compromised oxidative phosphorylation, and a malfunctioning electron transport chain (46). These shortcomings give rise to a lower mitochondrial membrane potential, heightened ROS generation, and the initiation of apoptotic pathways like caspase-3 (47). Moreover, psychological strain disturbs mitochondrial energy production within the hippocampus, curbing metabolic adaptability and weakening resilience against stress (48).

## 4 Herbal antidepressant supplements: an overview

Herbal and plant-derived supplements have garnered significant scholarly interest as adjunctive or alternative approaches for addressing depressive disorders. These natural compounds frequently exhibit multi-faceted mechanisms of action, encompassing the modulation of monoaminergic neurotransmission, augmentation of neurotrophic signaling pathways, anti-inflammatory properties, antioxidant functions, and regulation of the HPA axis. In contrast to traditional antidepressants that predominantly target a singular pathway, herbal supplements may offer more comprehensive neurobiological advantages that correspond with the complex etiology of depressive conditions (49). Numerous botanical species have been rigorously investigated for their potential antidepressant effects, including *Rhodiola rosea*, *Ginkgo biloba*, *Withania somnifera*, *Hypericum perforatum*, and *Crocus sativus*. A substantial number of these herbal remedies have a long-standing history within traditional medical systems such as traditional Chinese Medicine (TCM), Ayurveda, and European herbal traditions, and their therapeutic efficacy has been corroborated by contemporary preclinical and clinical research (50).

Herbal supplements typically exhibit a commendable safety profile and are generally well tolerated; however, it is imperative to consider potential interactions with conventional medications or anticoagulants (51). Standardized extracts and preparations subjected to quality control provide more reliable pharmacological effects in comparison to raw or non-standardized herbal products. From a mechanistic perspective, herbal antidepressants frequently modulate monoaminergic systems by either inhibiting MAO-A/B or augmenting the availability of serotonin, norepinephrine, and dopamine. These agents can also regulate hyperactivity of the HPA axis, enhance neuroplasticity and BDNF expression, mitigate neuroinflammation and oxidative stress, and safeguard mitochondrial function, thus improving cellular bioenergetics. Such multimodal effects render herbal supplements particularly appealing for integrative approaches in mental health, particularly in cases of stress-related or treatment-resistant depression (52). Notwithstanding the encouraging evidence, obstacles such as variability in extract composition, inadequate standardization, and a paucity of large-scale randomized clinical trials persist. Nonetheless, an increasing body of research substantiates the efficacy of herbal antidepressants as promising adjuncts or alternatives to pharmacotherapy, with the potential to enhance treatment outcomes through converging biological mechanisms.

## 5 Rhodiola rosea

### 5.1 Phytochemical composition

*Rhodiola rosea*, commonly referred to as golden root or Arctic root, is a perennial herb that is extensively utilized in traditional medicine due to its adaptogenic and mood-enhancing characteristics. The pharmacological properties of this herb are primarily ascribed to a diverse range of bioactive constituents, which include phenylpropanoids (such as rosavin, rosarin, and rosin), salidroside, tyrosol, flavonoids, tannins, and essential oils (53). Notably, rosavin and salidroside are regarded as key marker compounds for standardization, with salidroside demonstrating significant neuroprotective and antidepressant effects in both preclinical and clinical research. The presence of flavonoids and phenolic acids further contributes to the herb’s antioxidant and anti-inflammatory properties, thereby augmenting its overall adaptogenic efficacy (54).

### 5.2 Mechanisms of action

**Figure 1:**
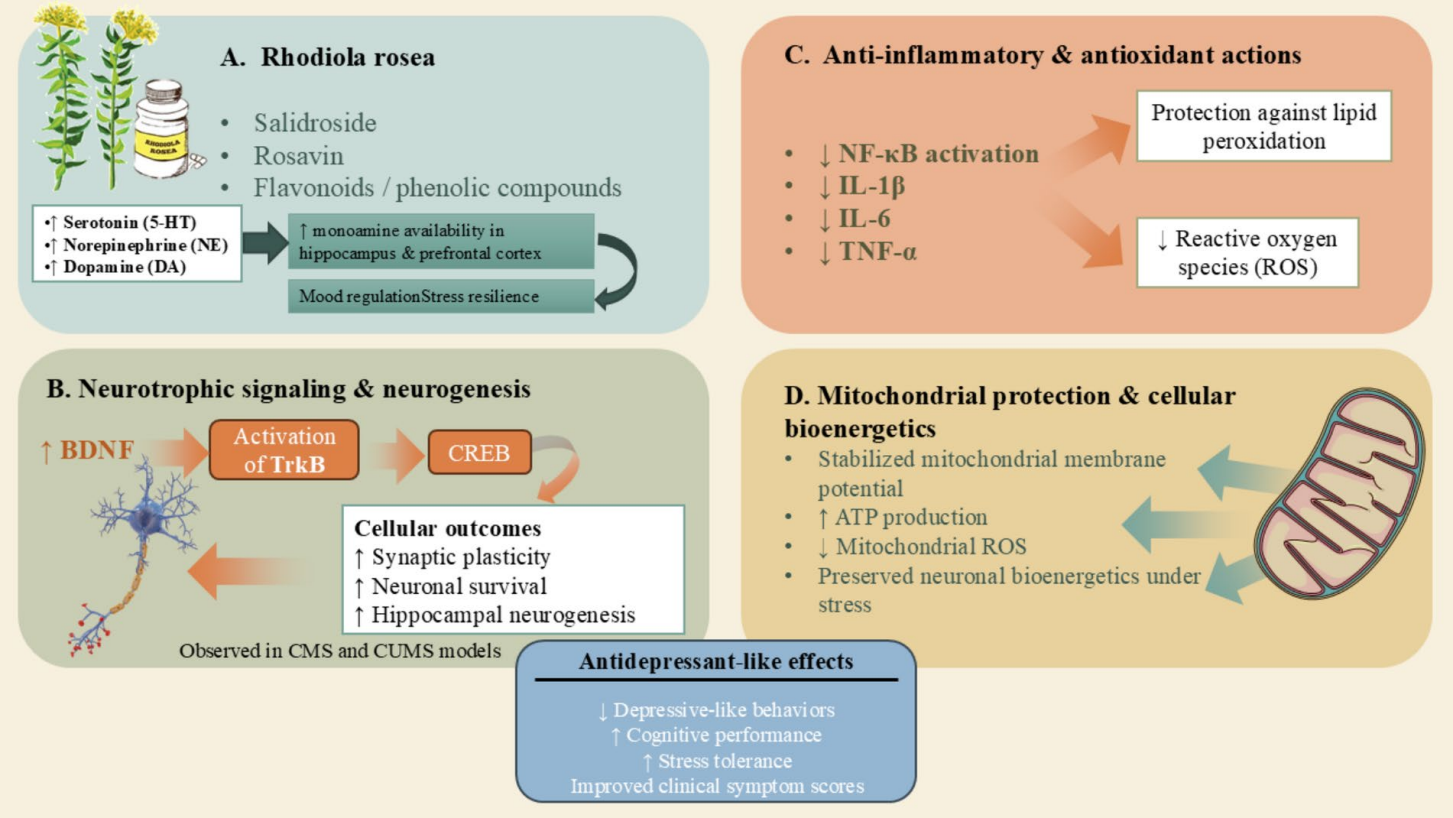
Multimodal antidepressant mechanisms of *Rhodiola rosea* in depressive disorders. This schematic illustrates the converging molecular and cellular mechanisms through which *Rhodiola rosea* exerts antidepressant-like effects. **(A)** Bioactive constituents of *R. rosea*, including salidroside, rosavin, and flavonoid/phenolic compounds, enhance monoaminergic neurotransmission by increasing the availability of serotonin (5-HT), norepinephrine (NE), and dopamine (DA) in key brain regions involved in mood regulation, thereby promoting stress resilience. **(B)** *R. rosea* activates neurotrophic signaling pathways, notably upregulating brain-derived neurotrophic factor (BDNF) and downstream TrkB-CREB signaling, which supports synaptic plasticity, neuronal survival, and hippocampal neurogenesis, as observed in chronic mild stress (CMS) and chronic unpredictable mild stress (CUMS) models. **(C)** Anti-inflammatory and antioxidant actions of *R. rosea* include suppression of NF-κB signaling and reduced production of pro-inflammatory cytokines (IL-1β, IL-6, TNF-α), alongside attenuation of reactive oxygen species (ROS) and lipid peroxidation, thereby limiting neuroinflammatory and oxidative damage. **(D)** *R. rosea* confers mitochondrial protection by stabilizing mitochondrial membrane potential, reducing mitochondrial ROS generation, and enhancing ATP production, preserving neuronal bioenergetics under stress conditions. Collectively, these multimodal actions converge to produce antidepressant-like effects, manifested as reduced depressive-like behaviors, improved cognitive performance, and enhanced stress tolerance.

The antidepressant properties of *Rhodiola rosea* are characterized by a multi-faceted mechanism of action, indicative of its extensive phytochemical composition (Figure 1). This botanical agent influences the monoaminergic system by elevating the levels of serotonin, norepinephrine, and dopamine in critical cerebral regions associated with mood regulation, including the hippocampus and the prefrontal cortex (55). In addition to its effects on monoamines, *R. rosea* demonstrates neurotrophic capabilities by enhancing the expression of BDNF and facilitating the activation of downstream TrkB–CREB signaling pathways, which are essential for synaptic plasticity, neuronal survival, and neurogenesis (56). Its anti-inflammatory mechanisms are enacted through the downregulation of pro-inflammatory cytokines such as IL-1β, IL-6, and TNF-α, along with the inhibition of NF-κB signaling, thus alleviating neuroinflammation that is implicated in the etiology of depressive disorders (54). Furthermore, *R. rosea* possesses antioxidant and mitochondrial-protective properties, effectively scavenging reactive oxygen species (ROS), stabilizing mitochondrial membrane potential, and augmenting adenosine triphosphate (ATP) synthesis, which collectively uphold neuronal bioenergetics during conditions of physiological stress (57).

### 5.3 Preclinical and clinical evidence

*Rhodiola rosea* has attracted considerable scholarly interest owing to its proposed antidepressant and adaptogenic properties. Preclinical investigations illustrate that its bioactive constituents may elevate monoamine concentrations, mitigate stress-induced neuroinflammatory responses, and stimulate neurotrophic factors such as BDNF, thereby facilitating neuronal survival and plasticity. Animal models designed to simulate depression and chronic stress consistently exhibit enhancements in mood, cognitive performance, and resilience subsequent to Rhodiola supplementation. Emerging clinical investigations involving human participants suggest that *Rhodiola rosea* may alleviate depressive manifestations, fatigue, and anxiety associated with stress, frequently presenting with a reduced incidence of adverse effects in comparison to traditional antidepressants. These observations accentuate the translational significance of *Rhodiola rosea* and emphasize its potential role as an adjunctive intervention in the treatment of depressive disorders, particularly among individuals experiencing stress-related or treatment-resistant symptoms. A proof-of-concept study explored the safety and effectiveness of *Rhodiola rosea* (*R. rosea*) in comparison to sertraline for individuals experiencing mild to moderate major depressive disorder. A total of 57 participants were randomly assigned to a 12-week regimen of standardized *R. rosea* extract (340 mg standardized to a content of rosavin 3.07%/rhodioloside 1.95%), sertraline (50 mg HCI), or a placebo. The assessments of depression (HAM-D, BDI, CGI/C) revealed modest, non-significant declines across all cohorts, with sertraline exhibiting a slightly more pronounced drop in HAM-D scores (−8.2) than *R. rosea* (−5.1) and placebo (−4.6). Although the likelihood of improvement was greater with sertraline compared to *R. rosea*, adverse events were reported more often in the sertraline cohort (63.2%) than in the *R. rosea* group (30%) and placebo (16.7%). These results indicated that *R. rosea* is better tolerated and may present a more favorable risk–benefit ratio, even with its marginally lower effectiveness (58) (Table 1). Another proof-of-concept trial, meticulously designed as a randomized, double-blind, placebo-controlled study, scrutinized the safety and effectiveness of Rhodiola capsules among 100 individuals grappling with mild to moderate major depressive disorder over a span of 12 weeks. Participants were carefully divided into three distinct groups: a high-dose Rhodiola group (0.6 g/day), a low-dose Rhodiola group (0.3 g/day), and a control group. Throughout the course of the study, depression metrics (HAM-D, BDI, CGI/C) were diligently monitored and evaluated. Remarkable enhancements were noted across all groups, with the most significant declines in HAM-D, BDI, and CGI scores occurring within the high-dose Rhodiola cohort when compared to both the low-dose and control groups. Both dosing regimens of Rhodiola proved effective in mitigating depressive symptoms and uplifting overall quality of life, with the higher doses showcasing a markedly superior antidepressant effect throughout the 12-week journey (59).

**Table 1:** Preclinical and clinical evidence on the antidepressant effects of *Rhodiola rosea* and its combinations: mechanisms and efficacy.

Type of evidence	Design & Participants	Intervention	Duration	Results	Conclusion	Reference
Clinical	57 participants; mild–moderate MDD; randomized placebo-controlled	*R. rosea*, sertraline, placebo	12 weeks	HAM-D: sertraline ↓↓; *R. rosea* ↓; placebo ↓ BDI: sertraline ↓; *R. rosea* ↓; placebo ↓ CGI/C: sertraline ↓; *R. rosea* ↓; placebo ↓	*R. rosea* less effective but better tolerated; favorable risk–benefit ratio for mild–moderate MDD	(58)
Clinical	100 patients; mild–moderate MDD; randomized double-blind placebo-controlled	Group A: sertraline + placebo; Group B: sertraline + 0.6 g/day Rhodiola; Group C: sertraline + 0.3 g/day Rhodiola	12 weeks	HAM-D: Group B ↓↓; Group C ↓; Group A ↓ BDI: Group B ↓↓; Group C ↓; Group A ↓ CGI: Group B ↓↓; Group C ↓; Group A ↓	Rhodiola capsules show dose-dependent antidepressant effects; high doses better than low doses; improved clinical symptoms and quality of life	(59)
Clinical	Phase III; 89 patients; mild–moderate depression; randomized double-blind placebo-controlled	Group A: 340 mg/day SHR-5; Group B: 680 mg/day SHR-5; Group C: placebo	6 weeks	HAM-D: Group A ↓; Group B ↓↓; Group C → BDI: Group A ↓; Group B ↓↓; Group C →	SHR-5 extract shows anti-depressive efficacy at 340–680 mg/day over 6 weeks; safe and well-tolerated	(60)
Preclinical	70 male Sprague–Dawley rats; CMS-induced depression	Low (1.5 g/kg), Medium (3 g/kg), High (6 g/kg) *Rhodiola rosea* extract	3 weeks	5-HT: ↑↑ (all doses restored to normal) Cell proliferation: ↑ (low dose normalized) Neuron quantity: ↑ (low dose repaired hippocampal neurons)	*Rhodiola rosea* extract improves 5-HT levels; low dose induces neural stem cell proliferation and hippocampal neuron repair	(61)
Clinical	Observational study; 45 adults with mild–moderate depression	Combination: Rhodiola 154 mg + Saffron 15 mg, 2 tablets/day	6 weeks	HAM-D: ↓↓ (58% reduction) HADS-anxiety & depression: ↓ (significant reduction) CGI/PGIC: ↑ (clinical improvement)	Combination of Rhodiola and Saffron significantly improves depressive and anxiety symptoms; safe and well-tolerated	(62)
Preclinical	Rats; CUMS-induced depression	Herba Rhodiolae (0.04 g/kg), Venlafaxine (0.035 g/kg), Rhodiola + GSK-3β inhibitor	—	Depression-like behaviors: ↓ (all treatments) Glutamate abnormalities: ↓ BDNF/TrkB signaling: ↑ (most with Rhodiola + GSK-3β inhibitor) GSK-3β expression: ↓	Rhodiola alleviates depressive-like behaviors via activation of BDNF/TrkB-GSK-3β pathway; combination with GSK-3β inhibitor most effective	(56)
Clinical	Double-blind, placebo-controlled study; adults with moderate depression (HAM-D 17–23); *n* = 62 non-students	Rhodiola 308 mg + Saffron 30 mg, 2 tablets/day	6 weeks	HAM-D: ↓↓ (10–12 point reduction) HADS-anxiety: ↓ HADS-depression: ↓ CGI-S/I: ↑ (improvement)	Combination improved psychological well-being in non-students; significant reduction in depression and anxiety; poor results in students	(63)
Preclinical	50 rats; CMS-induced depression	*Rhodiola rosea* (dose not specified)	3 weeks	5-HT: ↑↑ Cell proliferation: ↑↑ Cell differentiation: ↑↑ Neuron quantity: ↑↑	*Rhodiola rosea* enhances 5-HT, promotes neural stem cell proliferation/differentiation, and repairs hippocampal neurons in depressive rats	(64)

Increase (↑), no significant change (→), ↓ = modest decrease (improvement), ↓↓ = larger decrease (greater improvement).

MDD, major depressive disorder; HAM-D, Hamilton Depression Rating Scale; BDI, Beck Depression Inventory; CGI/C, Clinical Global Impression-Change; CGI-S, Clinical Global Impression-Severity; CGI-I, Clinical Global Impression-Improvement; PGIC, Patient Global Impression of Change; HADS, Hospital Anxiety and Depression Scale; CMS, chronic mild stress; CUMS, chronic unpredictable mild stress; 5-HT, 5-Hydroxytryptamine (serotonin); BrdU, bromodeoxyuridine; BDNF, brain-derived neurotrophic factor; TrkB, tropomyosin receptor kinase B; GSK-3β, glycogen synthase kinase 3 beta; SHR-5, standardized *Rhodiola rosea* extract SHR-5.

A phase III randomized, double-blind, placebo-controlled investigation assessed the therapeutic efficacy and safety profile of the standardized *Rhodiola rosea* extract SHR-5 in individuals diagnosed with mild to moderate depressive disorders over a duration of 6 weeks. Adult participants, aged between 18 and 70 years, exhibiting HAMD scores ranging from 21 to 31, were allocated to receive either a low-dose of SHR-5 (340 mg/day), a high-dose of SHR-5 (680 mg/day), or a placebo (60). The severity of depressive symptoms was evaluated utilizing HAMD and BDI scoring metrics at both baseline and on day 42 of the trial. Both groups receiving SHR-5 demonstrated statistically significant enhancements in overall depressive symptoms, sleep disturbances, emotional dysregulation, and somatic complaints, whereas the placebo cohort exhibited no such clinical improvements. No severe adverse reactions were documented throughout the trial period. These findings suggested that SHR-5 is both efficacious and well-tolerated, with the administration of higher doses correlating with enhanced antidepressant outcomes over the six-week period (60). A preclinical investigation examined the impact of *Rhodiola rosea* extract on serotonin concentrations, cellular proliferation, and the quantity of neurons within the hippocampus of depressive rats subjected to chronic mild stress (CMS). A total of 70 male Sprague–Dawley rats were systematically allocated into seven distinct groups, inclusive of control and three dosage categories of Rhodiola extract (1.5, 3, and 6 g/kg). Following a treatment duration of 3 weeks, hippocampal 5-HT concentrations were quantified through high-performance liquid chromatography, while neural proliferation was evaluated using BrdU labeling techniques. The results indicated that all groups administered Rhodiola exhibited a restoration of 5-HT levels to baseline conditions; notably, the low-dose extract (1.5 g/kg) specifically facilitated the proliferation of hippocampal neural stem cells and neuronal regeneration. These outcomes exhibited that Rhodiola extract has the potential to enhance serotonergic signaling pathways and promote neurogenesis, thereby contributing to the reparative processes within the hippocampus in models of depression (61).

An observational study investigated a unique blend of *Rhodiola rosea* and *Crocus sativus* (saffron) in a group of 45 adults experiencing mild to moderate depression. Participants were administered two tablets each day (containing 154 mg of rhodiola and 15 mg of saffron per tablet) for a duration of 6 weeks. Following the treatment period, the scores on the Hamilton Depression Rating Scale plummeted by an impressive 58% on average, with 85% of individuals noting a positive change. Remarkable decreases in both anxiety and depression scores were noted as quickly as 2 weeks into the study. Clinical evaluations from the patients and general practitioners alike revealed significant enhancements in depressive symptoms. This combination was well-accepted, with no serious side effects reported. These findings demonstrated that the duo of rhodiola and saffron might be a potent remedy for alleviating symptoms of depression and anxiety, although further placebo-controlled trials are necessary to validate the effectiveness (62). Depression, a widespread mental health challenge, frequently demonstrates minimal effectiveness with traditional antidepressants, underscoring the demand for innovative treatments. In an investigation, rats were exposed to a prolonged and unpredictable mild stress (CUMS) to evoke behaviors reminiscent of depression. The interventions encompassed Herba Rhodiolae (0.04 g/kg), venlafaxine (0.035 g/kg), and a synergistic approach combining Herba Rhodiolae with a GSK-3β inhibitor (10 μM). Behavioral assessments revealed that all interventions mitigated depressive-like manifestations, with the combined therapy proving to be the most potent. On a mechanistic level, Herba Rhodiolae amplified BDNF/TrkB signaling, while the GSK-3β inhibitor curtailed GSK-3β expression. This combination therapy effectively diminished abnormalities in the glutamate system and rectified autophagy disruptions. These data showed that Herba Rhodiolae, especially when paired with GSK-3β inhibition, showcases antidepressant properties by orchestrating BDNF/TrkB-GSK-3β signaling and neuroprotective mechanisms (56).

A clinical investigation was conducted to assess the therapeutic efficacy and safety profile of a synergistic formulation comprising *Rhodiola rosea* and *Crocus sativus* (saffron) in a cohort of 126 adults diagnosed with moderate depressive disorder over a duration of 6 weeks. Participants were administered two tablets daily (containing 308 mg of rhodiola and 30 mg of saffron) or a placebo control. Within the non-student demographic, the group receiving the supplement demonstrated a statistically significant decline in Hamilton Rating Scale for Depression (HAM-D) scores, achieving a reduction of 10 points at 3 weeks and 12 points at 6 weeks, alongside enhanced remission rates and superior improvements in both anxiety and depression metrics compared to the placebo group. No statistically significant effects were discerned within the student subset. Adherence to the treatment regimen and overall tolerability were deemed satisfactory. These findings demonstrated that the combination of rhodiola and saffron can effectively ameliorate depressive symptoms in adults experiencing moderate depression, particularly when clinical evaluations are utilized in conjunction with quantitative assessment tools (63). A preclinical investigation examined the impact of *Rhodiola rosea* on hippocampal serotonin (5-HT) concentrations, as well as neural proliferation, differentiation, and neuron quantity in rats experiencing depression induced by chronic mild stress. A total of 50 rats were systematically assigned into five distinct groups, encompassing control and treatment cohorts that received Rhodiola or fluoxetine over a duration of 3 weeks. The assessment of hippocampal 5-HT concentrations was conducted utilizing high-performance liquid chromatography, while proliferating and differentiating cells were identified through labeling with BrdU and beta-tubulin III. In comparison to the control group, the cohort treated with Rhodiola exhibited restored 5-HT concentrations, an increased prevalence of BrdU-positive and double-labeled cells, as well as normalized neuron quantities. These outcomes suggested that *Rhodiola rosea* may facilitate serotonergic functionality, enhance the proliferation and differentiation of neural stem cells, and potentially contribute to the repair of compromised hippocampal neurons in models of depression (64). Overall, numerous preclinical and clinical investigations have scrutinized *Rhodiola rosea* (*R. rosea*) and its synergistic applications in the context of mild to moderate depressive disorders. Proof-of-concept studies have substantiated that *R. rosea* is both safe and well-tolerated, yielding modest antidepressant effects, with higher dosages correlating with enhanced efficacy. Phase III trials have validated that standardized extracts of *R. rosea* (SHR-5) lead to improvements in depressive symptoms, emotional regulation, and sleep quality, accompanied by a minimal incidence of adverse events. Preclinical research has indicated that *R. rosea* augments hippocampal serotonin levels, stimulates neural stem cell proliferation, and facilitates neuronal repair mechanisms. Observational and clinical investigations that incorporate *R. rosea* alongside *Crocus sativus* (saffron) have demonstrated rapid and significant reductions in scores pertaining to depression and anxiety, particularly among adults who are not students. Mechanistically, *R. rosea* operates through BDNF/TrkB signaling pathways, serotonergic modulation, and neuroprotective mechanisms, underscoring its potential as a safe and efficacious alternative or adjunctive treatment in the management of depression.

The clinical evidence for *Rhodiola rosea* is supported by multiple randomized, double-blind, placebo-controlled trials, including a phase III study evaluating the standardized extract SHR-5, which demonstrated statistically significant improvements in depressive symptoms with a favorable tolerability profile. Nevertheless, most clinical trials were limited by modest sample sizes and relatively short intervention periods (6–12 weeks), restricting conclusions regarding long-term efficacy and relapse prevention. Proof-of-concept comparisons with sertraline suggest a more favorable safety profile for *R. rosea* but indicate comparatively lower antidepressant potency. Preclinical studies provide robust mechanistic support, particularly through serotonergic modulation, BDNF/TrkB signaling, and hippocampal neurogenesis, although translational dose equivalence remains uncertain. Overall, the evidence base for *Rhodiola rosea* is mechanistically coherent and clinically promising, but larger, adequately powered head-to-head trials are needed to more clearly define its therapeutic positioning.

### 5.4 Toxicology, pharmacokinetics, and standardization of *Rhodiola rosea*

*Rhodiola rosea* is generally regarded as safe and well tolerated when administered at clinically relevant doses. Human trials employing standardized extracts (e.g., SHR-5) at doses ranging from 200 to 680 mg/day have reported a low incidence of adverse events, primarily mild and transient symptoms such as dizziness, dry mouth, or gastrointestinal discomfort. Importantly, no significant hepatotoxicity, nephrotoxicity, or cardiotoxicity has been observed in short- to medium-term studies (6–12 weeks). Long-term safety data remain limited; however, available evidence from repeated-dose animal studies and clinical observations suggests a favorable safety margin, with no evidence of dependence, tolerance, or withdrawal phenomena (6).

The pharmacokinetic profile of *Rhodiola rosea* is largely driven by its key bioactive constituents, particularly salidroside and rosavin. Salidroside exhibits relatively rapid absorption following oral administration and demonstrates measurable penetration across the blood–brain barrier, consistent with its documented central neuroprotective and antidepressant effects. Preclinical studies indicate that salidroside reaches peak plasma concentrations within 1–2 h and distributes to hippocampal and cortical regions. Although rosavin displays lower oral bioavailability, it may contribute synergistically through peripheral stress-modulating and MAO-inhibitory actions. Comprehensive human pharmacokinetic data remain scarce, highlighting the need for further investigation into tissue distribution, metabolism, and elimination kinetics (6).

Standardization represents a critical determinant of Rhodiola’s reproducibility and clinical efficacy. Most high-quality preparations are standardized to a rosavin-to-salidroside ratio of approximately 3:1, which reflects the phytochemical composition of *R. rosea* root extracts used in clinical trials. The SHR-5 extract is among the most extensively studied formulations and serves as a benchmark for quality control. Variability in plant origin, harvesting conditions, and extraction methods can significantly alter phytochemical profiles, underscoring the importance of standardized manufacturing practices for clinical and translational applications (6).

## 6 Ginkgo biloba

### 6.1 Active constituents

*Ginkgo biloba* represents one of the most extensively investigated phytotherapeutic agents, recognized for its neuroprotective and cognitive-enhancing attributes. The pharmacological mechanisms of *G. biloba* are predominantly ascribed to its standardized leaf extracts, which encompass flavonoids (quercetin, kaempferol, isorhamnetin), terpenoids (ginkgolides A, B, C, J, and bilobalide), organic acids, and polyphenols (65). Flavonoids are instrumental in providing significant antioxidant and free radical-scavenging capabilities, whereas ginkgolides and bilobalide are acknowledged for their neuroprotective, anti-apoptotic, and anti-inflammatory properties. Standardized extracts, such as EGb 761, are frequently employed in clinical investigations to guarantee uniform concentrations of these bioactive constituents.

### 6.2 Mechanisms of action

**Figure 2:**
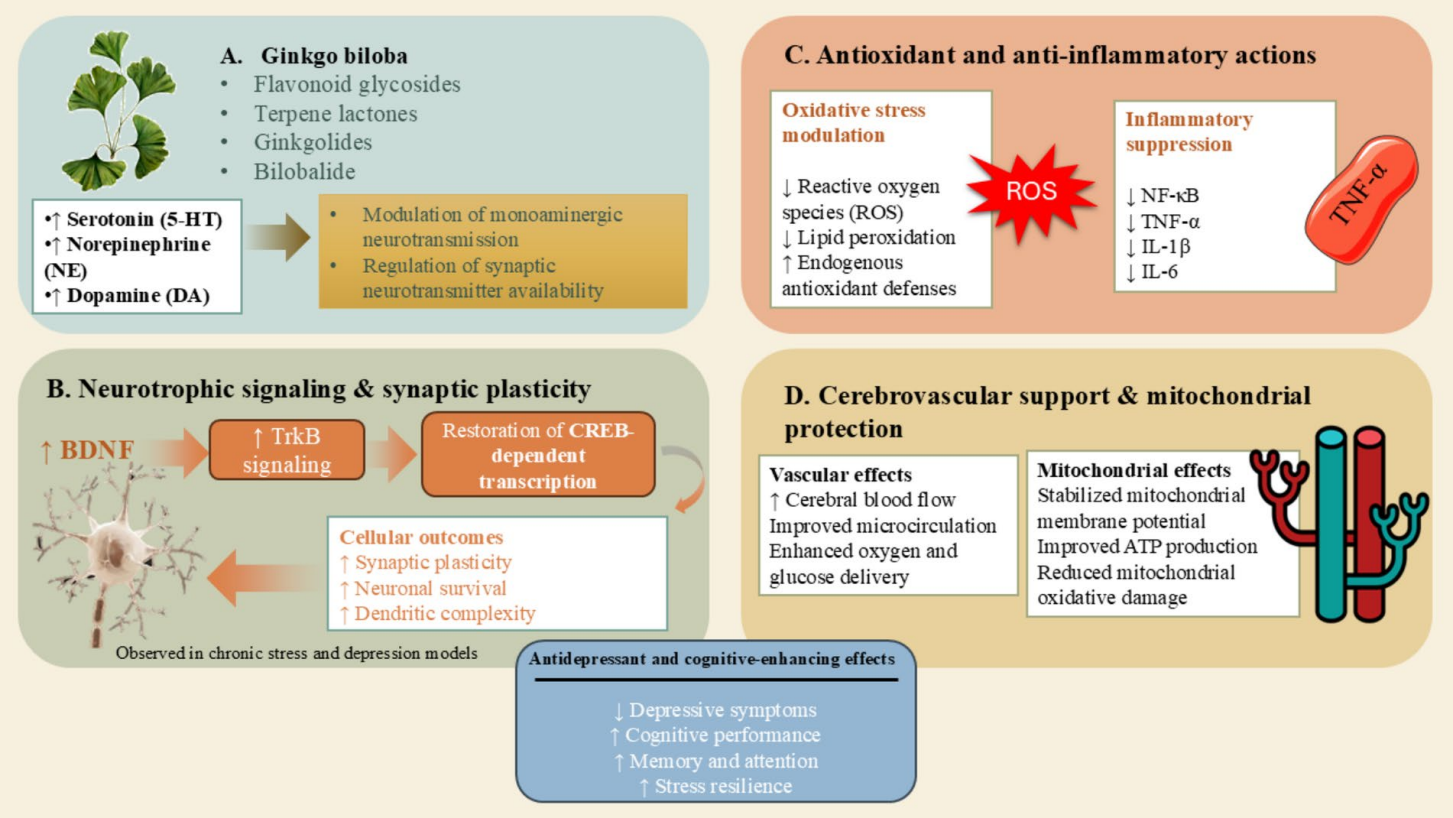
Mechanistic pathways underlying the antidepressant and neuroprotective effects of *Ginkgo biloba*. This figure illustrates the multifaceted biological mechanisms through which *Ginkgo biloba* exerts antidepressant and neuroprotective actions. Bioactive constituents—including flavonoids (quercetin, kaempferol, isorhamnetin) and terpenoids (ginkgolides A–J and bilobalide)—modulate monoaminergic neurotransmission by enhancing serotonergic, dopaminergic, and noradrenergic signaling. *Ginkgo biloba* promotes neurotrophic activity through the upregulation of brain-derived neurotrophic factor (BDNF), thereby improving synaptic plasticity, neuronal resilience, and neurogenesis. The extract reduces neuroinflammation by inhibiting NF-κB activation and decreasing pro-inflammatory cytokines such as IL-1β, IL-6, and TNF-α. Its flavonoid-rich antioxidant activity neutralizes reactive oxygen species and prevents lipid peroxidation, while its terpenoid components protect mitochondria by stabilizing mitochondrial membrane potential, increasing ATP production, and reducing cytochrome c release, collectively diminishing apoptosis and enhancing neuronal survival. Together, these mechanisms contribute to improved mood regulation, cognitive functioning, and neuroprotection in depressive pathology. **(A)** Active constituents of Ginkgo biloba. **(B)** Neurotrophic signaling & synaptic plasticity of Ginkgo biloba. **(C)** Antioxidant and anti-inflammatory actions of Ginkgo biloba. **(D)** Cerebrovascular support & mitochondrial protection of Ginkgo biloba.

The antidepressant and neuroprotective properties of *Ginkgo biloba* are attributable to its multifaceted mechanisms of action (Figure 2). It modulates the monoaminergic system by augmenting serotonergic, dopaminergic, and noradrenergic neurotransmission, thereby facilitating mood regulation and cognitive functioning (66). *G. biloba* further demonstrates neurotrophic effects through the upregulation of BDNF expression and the enhancement of synaptic plasticity, consequently promoting neuronal resilience and neurogenesis (67). The anti-inflammatory properties of *G. biloba* encompass the attenuation of pro-inflammatory cytokines, such as IL-1β, IL-6, and TNF-α, alongside the inhibition of NF-κB signaling pathways, which collectively mitigate neuroinflammation that is implicated in depressive pathology (10). Its antioxidant capabilities are instrumental in neutralizing reactive oxygen species (ROS), preventing lipid peroxidation, and safeguarding neuronal mitochondria, thereby enhancing energy metabolism and diminishing apoptosis. Moreover, *Ginkgo biloba* exerts protective effects on mitochondria by stabilizing the mitochondrial membrane potential, augmenting ATP production, and diminishing cytochrome c release, which collectively bolster neuronal survival in the face of stressors (10).

### 6.3 Preclinical and clinical evidence

A clinical investigation was conducted to assess the impact of *Ginkgo biloba* extract (EGb) as a supplementary treatment to citalopram in a cohort of 136 geriatric patients diagnosed with depression. The participants were stratified into two equal groups: one receiving EGb (Harbin HaoBo Pharmaceutical Co., Ltd. for treatment, 19.2 mg per time, and 3 times a day) in conjunction with citalopram (20 mg per day) and the other receiving citalopram exclusively. The severity of depression (measured by HAMD), levels of anxiety (assessed by HAMA), cognitive capabilities (evaluated through WCST), and serum S100B concentrations were thoroughly examined. The group receiving the combination therapy exhibited a more rapid onset of antidepressant efficacy, marked enhancements in HAMD and HAMA scores, and superior cognitive performance, characterized by a reduction in errors and an increase in accurate classifications on the WCST. Serum S100B, which serves as a biomarker for brain injury, demonstrated a more pronounced reduction in the EGb + citalopram cohort and exhibited a correlation with levels of depression, anxiety, and cognitive impairments. These outcomes showed that EGb not only augments the antidepressant effects but also facilitates improvements in cognitive functioning and may contribute to the restoration of neurological function in older adult patients (68) (Table 2). Sleep disturbances coupled with cognitive deficits are prevalent among patients diagnosed with depression who are undergoing treatment with conventional antidepressants, thereby underscoring the necessity for supplementary therapeutic interventions. A preliminary open-label investigation assessed the impact of *Ginkgo biloba* extract (EGb LI 1370) on both sleep quality and cognitive function in a cohort of 16 inpatients receiving trimipramine (69). Out of these, eight participants were administered adjunctive EGb (240 mg/day) over a four-week duration, while the remaining eight continued solely with trimipramine monotherapy. Polysomnographic analysis indicated that EGb significantly enhanced sleep efficiency, diminished the frequency of awakenings, reduced the duration of stage 1 sleep and the density of REM sleep, and augmented both stage 2 and slow-wave non-REM sleep; these effects were notably reversed following the cessation of EGb. These findings exhibited that EGb may facilitate improvements in sleep continuity and non-REM sleep among individuals with depression, potentially via the modulation of corticotropin-releasing hormone (CRH) activity, thereby presenting a promising adjunctive approach for the management of sleep disturbances associated with depression (69).

**Table 2:** Preclinical and clinical evidence on the antidepressant effects of *Ginkgo biloba* and its combinations: mechanisms and efficacy.

Type of evidence	Population/Model	Duration	Intervention	Comparators	Result(s)	Conclusion(s)	Reference
Clinical	136 elderly patients with depression	12-week treatment period, with evaluations at multiple time points (2, 4, 6, 8, and 12 weeks)	EGb + Citalopram	Citalopram alone	Faster antidepressant onset; greater reductions in HAM-D and HAM-A scores; improved WCST performance; decreased serum S100B levels, with S100B changes correlated with symptom severity	A synergistic effect of EGb with antidepressant therapy and potential restoration of neurologic function in elderly patients with depression	(68)
Clinical	16 depressed inpatients	6 weeks of trimipramine monotherapy	Trimipramine + EGb (240 mg/day)	Trimipramine alone	Increased sleep efficiency; reduced nocturnal awakenings; decreased stage 1 sleep and REM density; increased stage 2 and non-REM sleep; effects reversed after treatment discontinuation	EGb may enhance sleep continuity in depressed patients with sleep disturbance	(69)
Clinical	72 patients with SAP + depression	12-week	GBDP + standard therapy	Placebo + standard therapy	Improved angina stability; reduced angina frequency; improved SF-36 subscales; greater improvement in PCS; HAMD improved in both groups; good safety profile	No significant differences in adverse events between groups were observed, supporting the safety of GBDP in this population	(70)
Clinical	81 inpatients with major depression receiving ECT	2 weeks	Ginkgo pill 40 mg q8h	Placebo	Greater improvement in cognition and depression scores compared with placebo; significant improvements post-ECT; suggests antioxidant/flavonoid effects	Adjunctive *Ginkgo biloba* tablets improved cognitive status and reduced depressive symptoms compared with placebo in patients with major depression undergoing electroconvulsive therapy	(71)
Preclinical	Rat model induced by chronic stress	Exact number of weeks is not specified	EGb + venlafaxine	Untreated depressed rats	Increased BDNF; reduced nNOS; improved exploration behavior; reduced fecal output; synergistic neuroprotective effects with venlafaxine	EGb augments the neuroprotective and antidepressant effects of venlafaxine	(72)

HAMD, Hamilton Depression Rating Scale; HAMA, Hamilton Anxiety Rating Scale; WCST, Wisconsin Card Sorting Test; EGb, *Ginkgo biloba* extract; Cit, Citalopram; SAQ, Seattle Angina Questionnaire; SF-36, 36-Item Short-Form Health Survey; PCS, Physical Component Summary; MMSE, Mini-Mental State Examination; BDNF, brain-derived neurotrophic factor; nNOS, neuronal nitric oxide synthase.

Stable angina pectoris (SAP) frequently coexists with depressive disorders, which adversely impacts both prognosis and overall quality of life. A randomized, double-blind, multicenter clinical trial investigated the efficacy and safety of *Ginkgo biloba* dropping pill (GBDP) as an adjunctive treatment to standard therapy in a cohort of 72 SAP patients presenting with depression over a duration of 12 weeks (70). The participants were administered either GBDP or a placebo (5 pills, three times a day) in conjunction with standard treatment. GBDP demonstrated an improvement in angina stability, exhibited a trend toward a reduction in weekly angina episodes, and significantly enhanced quality of life, as assessed through SF-36 scores, in comparison to baseline measurements. Both treatment groups experienced improvements in depressive symptoms (as indicated by HAMD scores), with no statistically significant difference observed in the incidence of adverse events. These findings implied that GBDP, when utilized alongside conventional therapy, effectively alleviates angina symptoms, improves quality of life, and may contribute to a reduction in depressive symptoms among patients with SAP (70). A clinical trial was conducted to evaluate the impact of *Ginkgo biloba* on cognitive function and depressive symptoms among 81 inpatients undergoing bilateral electroconvulsive therapy (ECT) for the treatment of major depressive disorder. Participants were systematically randomized to receive either *Ginkgo biloba* capsules (40 mg administered every 8 h) or a placebo throughout the two-week duration of ECT. Cognitive function was assessed utilizing the Mini-Mental State Examination, while the severity of depression was measured employing the Hamilton Depression Rating Scale at both pre- and post-ECT intervals. Both treatment cohorts exhibited significant enhancements in cognitive performance and depressive symptoms; however, the group receiving *Ginkgo biloba* exhibited more pronounced improvements in both cognitive and affective domains. These outcomes showed that *Ginkgo biloba*, potentially attributable to its flavonoid and antioxidant constituents, may facilitate cognitive recuperation and ameliorate depressive symptoms in individuals undergoing ECT (71).

A preclinical investigation examined the impacts of *Ginkgo biloba* extract (EGb) and venlafaxine on depression elicited by chronic and multifaceted stressors in a rat model (72). Exposure to stress resulted in diminished hippocampal brain-derived neurotrophic factor (BDNF) expression in the CA3 region of neurons, a decrease in exploratory behavior, an increase in fecal output, and heightened levels of neuronal nitric-oxide synthase (nNOS), all of which are indicative of neural impairment (72). Administration of EGb (40 mg/kg) and venlafaxine (15 mg/kg) reinstated BDNF expression, enhanced exploratory behavior, and lowered nNOS levels as well as stress-related physiological responses. These results imply that EGb, particularly when utilized in conjunction with venlafaxine, confers neuroprotective properties, alleviates stress-induced neural damage, and mitigates depressive-like behaviors. This underscores the potential of EGb as a complementary therapeutic approach to augment neuronal safeguarding while concurrently diminishing the adverse effects associated with traditional antidepressant treatments (72). Totally, few clinical and preclinical studies have demonstrated the antidepressant and neuroprotective potential of *Ginkgo biloba* (EGb). In elderly patients with depression, EGb combined with citalopram accelerated therapeutic onset, improved depressive and anxiety symptoms, enhanced cognitive performance, and reduced serum S100B, a marker of neuronal injury. EGb also improved sleep quality in depressed inpatients by increasing sleep efficiency, enhancing non-REM slow-wave sleep, and reducing awakenings, likely via modulation of CRH activity. Among patients with stable angina pectoris and comorbid depression, adjunctive GBDP improved angina stability, quality of life, and depressive symptoms. *Ginkgo biloba* further enhanced cognition and mood in patients undergoing ECT and, in rat models of stress-induced depression, mitigated neural damage, restored hippocampal BDNF, and reduced nNOS, highlighting its role as a safe and effective adjunctive therapy for depression and neuroprotection.

The evidence supporting *Ginkgo biloba* in depression is characterized by heterogeneity in study design and clinical application, with most trials evaluating its role as an adjunctive rather than a stand-alone antidepressant. Randomized and controlled studies indicate beneficial effects on depressive symptoms, sleep quality, cognitive function, and neurobiological markers when *Ginkgo biloba* is combined with conventional antidepressants or cardiovascular therapies. However, variability in formulations, dosing regimens, and outcome measures limits direct cross-study comparability. Preclinical investigations consistently demonstrate neuroprotective, anti-inflammatory, and monoaminergic effects, strengthening biological plausibility. Despite these mechanistic strengths, the paucity of large-scale monotherapy trials and limited long-term outcome data constrain definitive conclusions regarding its independent antidepressant efficacy.

### 6.4 Toxicology, pharmacokinetics, and standardization of *Ginkgo biloba*

Although *Ginkgo biloba* leaf extract is widely perceived as safe due to its natural origin, the article emphasizes that its toxicological profile is not fully understood, particularly for long-term use. Human epidemiological data linking GBE to toxicity or cancer risk are scarce, partly because U.S. regulations do not require manufacturers of dietary supplements to submit toxicity data. As a result, adverse events may be underreported (73). Standardized *Ginkgo biloba* leaf extracts generally exhibit low acute and sub-chronic toxicity when used at recommended doses. Animal studies show high LD₅₀ values, suggesting a wide margin of safety. However, adverse effects such as gastrointestinal discomfort, headache, dizziness, and allergic skin reactions have been reported in humans. Of particular concern is the potential for increased bleeding risk, attributed mainly to ginkgolides’ platelet-activating factor (PAF) antagonism. The presence of ginkgolic acids is associated with cytotoxicity and allergenicity, underscoring the need to limit their concentration in commercial products (74).

Pharmacokinetic data indicate that the major active constituents of *Ginkgo biloba*, including flavonoids and terpene lactones (ginkgolides A, B, C, and bilobalide), are absorbed following oral administration, though their bioavailability varies. Terpene lactones are relatively well absorbed and can cross the blood–brain barrier, supporting their proposed neurological effects. Metabolism occurs primarily in the liver, with elimination via urine and bile. Variability in pharmacokinetic profiles among individuals, may influence clinical efficacy and safety, particularly when *Ginkgo biloba* is taken concomitantly with other medications (74).

In addition, ginkgolic acids and related alkylphenols, naturally present in ginkgo leaves, are strongly allergenic and cytotoxic. These compounds can induce severe contact dermatitis and immunotoxic effects, similar to poison ivy reactions. Although standardized extracts are processed to reduce ginkgolic acid content to below 5 ppm, variability among commercial products may still pose safety risks. The presence of minor constituents such as 2-hexenal, an α,β-unsaturated aldehyde capable of forming DNA adducts, which may contribute to carcinogenic risk when exposure is chronic (73).

Standardization is critical for ensuring the safety, efficacy, and reproducibility of *Ginkgo biloba* preparations. Most clinical studies rely on standardized extracts such as EGb 761, typically containing approximately 24% flavonoid glycosides and 6% terpene lactones, with ginkgolic acids restricted to minimal levels. Without proper standardization, significant variability in chemical composition can occur, leading to inconsistent therapeutic outcomes and increased risk of adverse effects (74).

## 7 Ashwagandha (*Withania somnifera*)

### 7.1 Bioactive components

Ashwagandha, a prominent adaptogenic herb utilized in the context of Ayurvedic medicine, encompasses a wide spectrum of bioactive phytochemicals that form the basis of its therapeutic efficacy. The principal active ingredients are withanolides, a class of steroidal lactones, which are regarded as the primary agents responsible for its neuroprotective and psychotropic effects (75). Additionally, other significant constituents comprise alkaloids, saponins, withanosides, and sitoindosides, which manifest antioxidant, anti-inflammatory, and anxiolytic properties (76). Withanolides function as pleiotropic compounds that modulate central nervous system signaling pathways, maintain redox equilibrium, and facilitate immune regulation. In preclinical investigations, withanolide A and withanone have demonstrated substantial neuroregenerative capabilities, encompassing dendritic proliferation and synaptic reconstruction (77). The synergistic interplay among withanolides, sitoindosides, and saponins seemingly propels the adaptogenic attributes of Ashwagandha by modulating the responses of stress hormones, regulating neurotransmitter systems, and augmenting the expression of neurotrophic factors.

### 7.2 Mechanisms of action

Ashwagandha demonstrates antidepressant properties through the integration of monoaminergic, neurotrophic, and anti-inflammatory pathways, thereby establishing its potential as a natural adjunct in the management of mood disorders. A multitude of investigations suggest that Ashwagandha augments monoaminergic neurotransmission by elevating the concentrations of serotonin (5-HT), dopamine (DA), and norepinephrine (NE) within the central nervous system (78, 79). The withanolides present in Ashwagandha may influence monoamine oxidase (MAO) activity, which subsequently enhances the synaptic availability of monoamines. Preclinical research has indicated that *Withania somnifera* elicits antidepressant-like effects that are comparable to those of imipramine and fluoxetine, thereby corroborating its involvement in serotonergic and noradrenergic signaling cascades (78). Chronic inflammation constitutes a significant factor contributing to the etiology of depression, while Ashwagandha exhibits pronounced anti-inflammatory properties. Withaferin A exerts inhibitory effects on NF-κB, COX-2, and pro-inflammatory cytokines, including TNF-α, IL-1β, and IL-6 (80, 81). These mechanisms serve to diminish neuroinflammation and alleviate the inflammatory burden present in peripheral tissues. In preclinical models that simulate stress-induced depression, supplementation with Ashwagandha has been shown to markedly reduce the release of cytokines, restore antioxidant defenses (e.g., glutathione, superoxide dismutase), and lower markers indicative of oxidative stress (82). Through the concurrent modulation of inflammatory, oxidative, and neuroendocrine pathways, Ashwagandha effectively disrupts the positive feedback loop that exists between stress, inflammation, and compromised neurotransmission, thereby promoting mood stabilization. Both clinical and preclinical investigations have substantiated significant reductions in cortisol concentrations following the administration of Ashwagandha (83). Withanolides are known to engage with GABAergic and serotonergic pathways, which mitigates the hyperactivation of the HPA axis, resulting in enhanced stress tolerance and anxiolytic effects (78). Oxidative stress is fundamentally implicated in the etiology of depressive disorders. The herbal supplement Ashwagandha augments endogenous antioxidant mechanisms by elevating the activity of catalase, glutathione peroxidase, and superoxide dismutase, concomitantly diminishing the process of lipid peroxidation (84). Additionally, its protective effects on mitochondrial function further bolster neuronal energy metabolism and enhance resilience against damage precipitated by stress.

### 7.3 Preclinical and clinical evidence

A randomized controlled trial was conducted to assess the therapeutic efficacy and safety of Ayurvedic interventions, specifically tablet Brahmi vati 500 mg thrice a day and Liquid Aswagandarista 10 mL thrice a day, in comparison to escitalopram among 50 participants diagnosed with major depressive disorder over a duration of 60 days (85). The participants underwent evaluations at 15-day intervals utilizing the Hamilton Depression Rating Scale (HDRS), Hamilton Anxiety Rating Scale (HARS), Clinical Global Impression (CGI) scales, the Udvalg for Kliniske Undersøgelser (UKU) side effect scale, the World Health Organization Quality of Life-BREF (WHOQOL-BREF), the Pittsburgh Sleep Quality Index (PSQI), and the Brief Psychiatric Rating Scale (BPRS). Both cohorts exhibited statistically significant enhancements in the domains of depression, anxiety, psychotic manifestations, and overall clinical assessments. The Ayurvedic cohort revealed superior advancements in quality-of-life metrics and a reduced incidence of adverse effects, whereas the control group displayed improved sleep quality. The laboratory investigations, encompassing liver function assays and serum creatinine levels, consistently remained within standardized normal ranges. These results suggested that Brahmi vati and Aswagandarista may serve as effective and safe alternatives to traditional antidepressants, providing supplementary advantages in terms of quality of life and tolerability (85) (Table 3). A preclinical investigation assessed the antidepressant properties of Ashwagandha in adolescent rodents subjected to chronic unpredictable mild stress (CUMS). Depression-like behaviors were elicited, and the adolescent rats received either Ashwagandha (50 mg/kg/day) or sertraline (5.0 mg/kg/day) as treatment. Behavioral evaluations (forced swim, sucrose preference, and elevated plus maze) in conjunction with biochemical and histological assessments were employed to gauge the effects of the interventions (86). The CUMS paradigm resulted in an elevation of pro-apoptotic proteins (Bax, caspase-3) and inflammatory markers (TNF-α, IL-1β), concurrently leading to a decrease in BDNF and GFAP levels. Treatment with Ashwagandha effectively ameliorated these alterations, demonstrating superior efficacy compared to sertraline, and additionally mitigated stress-induced weight reduction. These data showed that Ashwagandha demonstrates antidepressant-like effects in models of adolescent depression by attenuating apoptosis, neuroinflammation, and enhancing neurotrophic factors, thereby underscoring its potential as a therapeutic agent for early intervention in adolescent depressive disorders (86).

**Table 3:** Preclinical and clinical evidence on the antidepressant effects of Ashwagandha: mechanisms and efficacy.

Type of evidence	Population/Design	Intervention	Controls	Duration	Result(s)	Conclusion	Reference
Clinical	50 adults with MDD (DSM-5); randomized controlled, 60-day intervention	Brahmi vati 500 mg TID + Aswagandharista 10 mL TID	Escitalopram 10 mg BID	60-day (approximately 8-week)	Ayurveda group showed significantly better WHOQOL-BREF and UKU scores; escitalopram better for PSQI; both groups improved significantly on HDRS, HARS, BPRS, CGI; labs remained normal	Ayurvedic treatment with Brahmi vati and Aswagandharista produced improvements in depressive symptoms comparable to escitalopram, with better quality-of-life scores and fewer side effects	(85)
Clinical	Adolescent rats exposed to chronic unpredictable mild stress	Ashwagandha extract	Escitalopram 10 mg BID	17-day	CUMS ↑ apoptosis & inflammation; ↓ BDNF & GFAP. Ashwagandha more effective than sertraline in reducing apoptosis & inflammation and restoring BDNF/GFAP; prevented weight loss	Ashwagandha treatment produced antidepressant-like effects in adolescent rats exposed to CUMS, reducing markers of neuronal apoptosis and neuroinflammation and improving behavioral measures compared with stressed controls, suggesting its potential utility in ameliorating depression-like states	(86)
Clinical	10 elderly patients with Avasada (depression)	Ashwagandha Siddha Tailadhara (Shirodhara with medicated oil)	None (single-arm pilot)	Not specified	Highly significant improvement (*p* < 0.0001) in key depressive symptoms and HDRS domains, especially insomnia; non-habit forming and well tolerated	Ashwagandha Siddha Tailadhara significantly improved depressive symptoms and sleep disturbances in elderly patients and was well tolerated, indicating its potential as a non-habit-forming intervention for depression in older adults	(87)
Clinical	70 adults with mild–moderate depression & anxiety; 90-day double-blind RCT	500 mg Ashwagandha extract (2.5% withanolides) + 5 mg piperine daily	Placebo	90-day (12-week)	ARE group showed significantly greater improvements (*p* < 0.001) in anxiety, depression, sleep quality, and QOL; serum serotonin increased (vs. decreased in placebo); well tolerated	Adjunctive treatment with a standardized *Withania somnifera* root extract (500 mg, 2.5% withanolides) plus piperine significantly improved depressive and anxiety symptoms, sleep quality, and quality of life compared with placebo	(88)

MDD, major depressive disorder; DSM-5, Diagnostic and Statistical Manual of Mental Disorders, Fifth Edition; RCT, randomized controlled trial; TID, three times daily; BID, twice daily; WHOQOL-BREF, World Health Organization Quality of Life-BREF; UKU, Udvalg for Kliniske Undersøgelser Side Effect Rating Scale; PSQI, Pittsburgh Sleep Quality Index; HDRS, Hamilton Depression Rating Scale; HARS, Hamilton Anxiety Rating Scale; BPRS, Brief Psychiatric Rating Scale; CGI, clinical global impression; CUMS, chronic unpredictable mild stress; BDNF, brain-derived neurotrophic factor; GFAP, glial fibrillary acidic protein; ARE, Ashwagandha root extract; QOL, quality of life.

A preliminary investigation assessed the therapeutic efficacy of Ashwagandha Siddha Tailadhara, an Ayurvedic Panchakarma intervention, in a cohort of 10 individuals diagnosed with Avasada (depression). Tailadhara encompasses the systematic infusion of medicated oil onto the forehead and is conventionally employed for the treatment of insomnia, cephalalgia, hypertension, and depressive disorders. Participants were evaluated for amelioration in subjective symptoms as well as modifications in Hamilton Depression Rating Scale (HDRS) scores. The intervention yielded markedly significant enhancements in fatigue, diminished motivation, and anhedonia, alongside notable improvements in sleep disturbances, particularly those occurring in the middle of the night and early morning insomnia. The procedure was administered with a high degree of tolerability, did not induce dependency, and exhibited both stress-relieving and tranquilizing properties. These findings indicated that Ashwagandha Siddha Tailadhara serves as a viable intervention for the management of depression in the elderly demographic (87). A randomized, double-blind, placebo-controlled investigation was conducted to assess the effects of standardized Ashwagandha root extract (ARE, 500 mg/day containing 2.5% withanolides and 5 mg piperine) on individuals experiencing mild to moderate depression and anxiety over a 90-day period involving 70 participants. The outcomes measured encompassed Hamilton Depression Rating Scale (HDRS) and Hamilton Anxiety Rating Scale (HARS) scores, sleep quality evaluated via the Global Sleep Quality Scale (GSQS), and overall quality of life (QOL). Supplementation with ARE resulted in statistically significant enhancements in depression, anxiety, sleep quality, and overall QOL in comparison to the placebo group at all assessed time points. Notably, serum serotonin levels exhibited an increase within the ARE group, in contrast to a decrease observed in the placebo cohort. The extract demonstrated a favorable safety profile, characterized by the absence of significant adverse events or laboratory abnormalities. These results substantiate the efficacy and safety of ARE in ameliorating mood, anxiety, and neurochemical equilibrium in individuals with mild to moderate depression (88). Overall, clinical and preclinical investigations suggest that Ayurvedic interventions, notably Brahmi vati, Aswagandarista, and Ashwagandha (root extract or Siddha Tailadhara), exhibit efficacy and safety in the management of depressive disorders. Brahmi vati and Aswagandarista demonstrated efficacy comparable to that of escitalopram, in conjunction with enhanced quality of life and diminished side effects. Ashwagandha has revealed antidepressant-like properties in adolescent models of depression through the attenuation of apoptosis, reduction of neuroinflammation, and promotion of neurotrophic factors. In geriatric populations, Ashwagandha Siddha Tailadhara has been shown to ameliorate mood, increase motivation, and improve sleep quality, whereas standardized Ashwagandha root extract has been associated with enhancements in depression, anxiety, sleep quality, and serotonin levels among adults experiencing mild to moderate depressive symptoms. Collectively, these findings lend credence to the therapeutic potential of Ashwagandha-based interventions as promising and well-tolerated alternatives or adjuncts in the treatment of depressive disorders.

**Figure 3:**
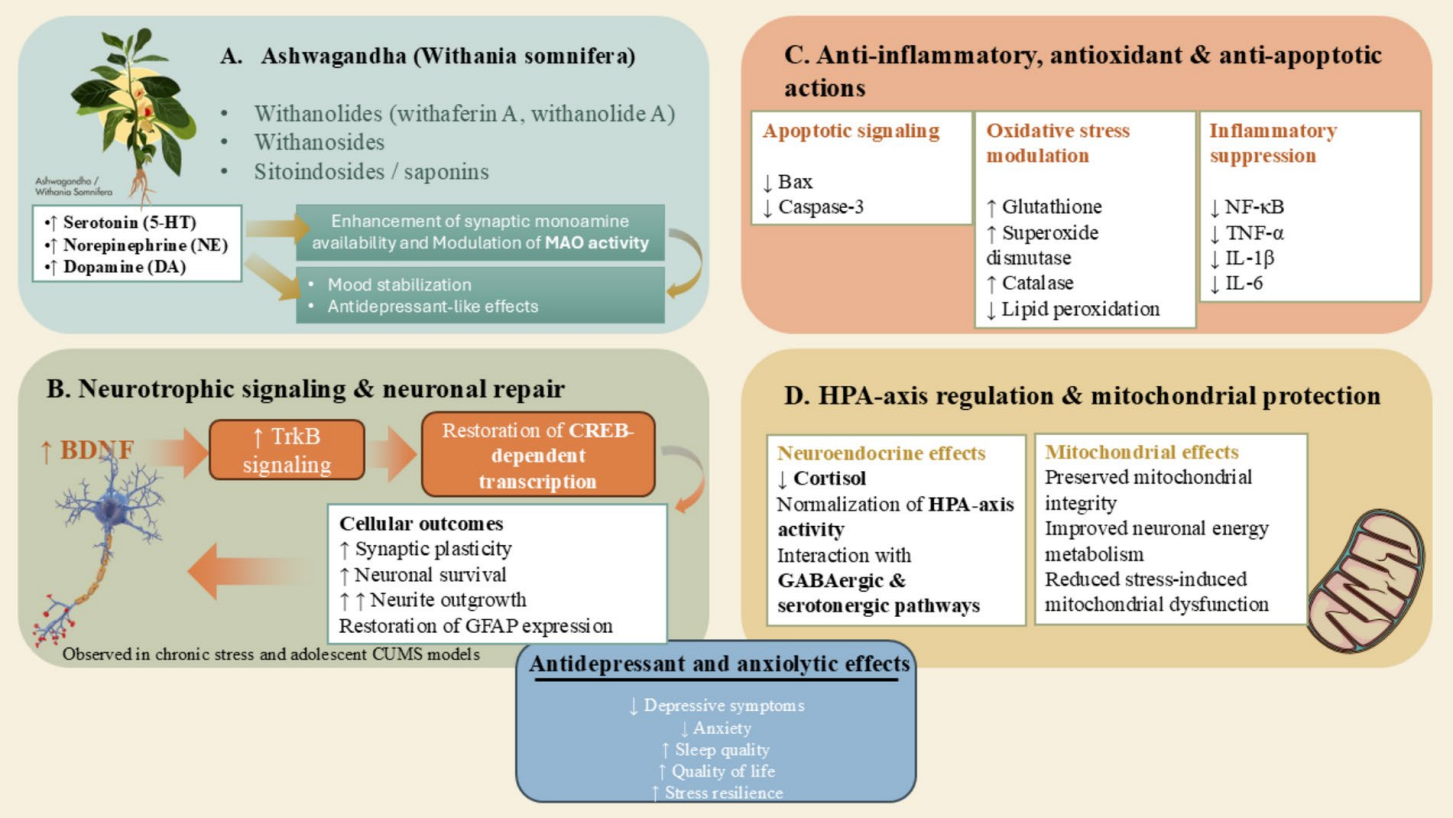
Multimodal antidepressant and adaptogenic mechanisms of *Withania somnifera* (Ashwagandha). This figure summarizes the interconnected molecular and cellular mechanisms underlying the antidepressant and adaptogenic effects of *Withania somnifera*. **(A)** Bioactive constituents of Ashwagandha, including withanolides (e.g., withaferin A and withanolide A), withanosides, and sitoindosides/saponins, modulate monoaminergic neurotransmission by increasing the availability of serotonin (5-HT), dopamine (DA), and norepinephrine (NE), in part through regulation of monoamine oxidase activity, contributing to mood stabilization and antidepressant-like effects. **(B)** Ashwagandha enhances neurotrophic signaling by upregulating brain-derived neurotrophic factor (BDNF) and activating TrkB-CREB-dependent transcription, thereby promoting synaptic plasticity, neuronal survival, neurite outgrowth, and neuronal repair, as demonstrated in chronic stress and adolescent chronic unpredictable mild stress (CUMS) models. **(C)** Anti-inflammatory, antioxidant, and anti-apoptotic actions of Ashwagandha include suppression of NF-κB signaling and reduced levels of pro-inflammatory cytokines (TNF-α, IL-1β, IL-6), enhancement of endogenous antioxidant defenses (glutathione, superoxide dismutase, catalase), attenuation of lipid peroxidation, and downregulation of pro-apoptotic markers such as Bax and caspase-3. **(D)** Ashwagandha regulates hypothalamic-pituitary-adrenal (HPA) axis activity by reducing cortisol levels and normalizing stress responses, while also preserving mitochondrial integrity, improving neuronal energy metabolism, and limiting stress-induced mitochondrial dysfunction. Collectively, these multimodal actions converge to produce antidepressant and anxiolytic effects, manifested as reduced depressive and anxiety symptoms, improved sleep quality and quality of life, and enhanced stress resilience.

Clinical evidence for Ashwagandha includes several randomized, double-blind, placebo-controlled trials demonstrating reductions in depressive and anxiety symptoms, improved sleep quality, and favorable tolerability, often accompanied by mechanistic signals such as increased serotonin levels and modulation of stress biomarkers. However, many studies were conducted in populations with mild to moderate symptoms and lacked active antidepressant comparators, limiting conclusions regarding relative efficacy. Additionally, heterogeneity in extract formulations, dosing strategies, and phytochemical standardization complicates clinical translation. Preclinical models consistently support antidepressant-like effects mediated through serotonergic, anti-inflammatory, and neuroprotective pathways, providing strong mechanistic rationale. Collectively, the evidence positions Ashwagandha as a promising stress-modulating and adjunctive intervention, though larger comparator-controlled trials are required to establish its role in major depressive disorder (Figure 3).

### 7.4 Phytochemistry, extraction advances, and pharmacological significance of *Withania somnifera* (Ashwagandha)

*Withania somnifera* (L.) Dunal, commonly known as Ashwagandha, is a widely used medicinal plant in traditional medicine systems and has attracted growing scientific interest due to its broad pharmacological potential. Native to arid and semi-arid regions of South Asia, Ashwagandha has been traditionally employed as a rejuvenating tonic and adaptogen (89). Contemporary research has expanded its relevance by elucidating its phytochemical composition, molecular mechanisms of action, and therapeutic significance in chronic and systemic disorders (89). The pharmacological activity of Ashwagandha is primarily attributed to a diverse range of bioactive constituents, among which withanolides constitute the most important class (90). Withanolides are naturally occurring steroidal lactones derived from an ergostane framework, and their structural diversity, arising from variations in oxidation, hydroxylation, and glycosylation, underlies their multitarget biological effects. Key compounds such as withaferin A, withanolide A, and withanosides have demonstrated antioxidant, anti-inflammatory, neuroprotective, immunomodulatory, and anticancer activities. In addition, alkaloids, flavonoids, phenolic compounds, and saponins contribute synergistically to the plant’s adaptogenic and therapeutic properties (89, 90).

Advances in extraction technologies have significantly improved the recovery and stability of Ashwagandha phytochemicals (91). While conventional aqueous and alcoholic extractions remain widely used, modern techniques such as supercritical fluid extraction, microwave-assisted extraction, and ultrasound-assisted extraction offer superior efficiency, enhanced selectivity for withanolides, and reduced solvent usage. These methods allow better preservation of thermolabile compounds and improve batch-to-batch consistency, which is essential for pharmaceutical and nutraceutical applications (89, 91).

Despite promising pharmacological profiles, the safety and toxicology of Ashwagandha warrant careful consideration. Acute and subchronic toxicity studies in rodents generally report high LD₅₀ values and no significant adverse effects at commonly used doses, indicating a wide safety margin. However, several investigations have documented dose-dependent effects on liver enzymes and histopathological changes in high-dose groups, suggesting potential hepatotoxicity under excessive or prolonged exposure. Reproductive toxicity studies in animal models have reported altered sperm parameters and effects on estrous cyclicity at supra-therapeutic doses (92). Human clinical trials typically report mild gastrointestinal disturbances and occasional allergic reactions, but rare cases of elevated liver enzymes have been noted, underscoring the need for cautious use, especially in individuals with preexisting liver conditions (92, 93).

## 8 Translational and clinical implications

The convergent yet distinct neurobiological actions of *Rhodiola rosea*, *Ginkgo biloba*, and *Withania somnifera* (Ashwagandha) provide a mechanistically informed framework for their rational translation into clinical practice. Rather than functioning as isolated antidepressant agents, these botanicals target complementary nodes within the interconnected pathophysiology of depressive disorders, encompassing monoaminergic dysregulation, impaired neurotrophic signaling, HPA-axis hyperactivity, neuroinflammation, oxidative stress, and mitochondrial dysfunction. This systems-level convergence supports their potential utility as adjunctive or phenotype-specific interventions within personalized treatment strategies (94, 95).

*Ginkgo biloba* exhibits a distinct translational profile, with antidepressant benefits emerging most clearly in populations with cognitive impairment, neurovascular compromise, or age-related vulnerability. Its ability to preserve mitochondrial integrity, enhance cerebral perfusion, mitigate oxidative stress, and support synaptic plasticity positions it as a neuroprotective adjunct rather than a primary monoaminergic antidepressant. Clinical evidence indicating accelerated antidepressant response, improved cognition, enhanced sleep architecture, and reduced biomarkers of neuronal injury in elderly and medically comorbid populations underscores its relevance in late-life depression and depression associated with cognitive or vascular dysfunction (96). Ashwagandha exhibits significant anxiolytic and antidepressant properties, particularly through its mechanisms involving the reduction of cortisol levels and the augmentation of brain-derived neurotrophic factor (BDNF) signaling, rendering it a compelling option for individuals experiencing mixed anxiety-depressive symptoms or elevated stress levels (83). Furthermore, these supplements may function as complementary interventions in conjunction with selective serotonin reuptake inhibitors (SSRIs) or serotonin-norepinephrine reuptake inhibitors (SNRIs), potentially enhancing therapeutic efficacy or mitigating the necessity for higher dosages (97). Nevertheless, the application of botanical extracts within clinical contexts necessitates a judicious approach. The heterogeneity in the standardization of botanical extracts, the potential for herb–drug interactions particularly involving anticoagulants in the context of *Ginkgo biloba*, and the scarcity of extensive randomized controlled trials underscore the imperative for precision-oriented methodologies in clinical decision-making (98). The careful selection of patients, biomarker-driven monitoring, and tailored dosing of supplements may contribute to the optimization of therapeutic results while concurrently mitigating associated risks. Ultimately, the incorporation of these botanical agents within a framework that is both evidence-based and personalized may foster resilience, enhance mood regulation, and support sustained mental well-being in individuals experiencing depressive disorders.

Importantly, the complementary mechanisms of these botanicals suggest potential value in stratified or adjunctive treatment frameworks rather than interchangeable use. *Rhodiola rosea* primarily targets stress-induced neurotransmitter imbalance, *Ginkgo biloba* supports neuronal and mitochondrial resilience, and Ashwagandha modulates neuroendocrine and inflammatory pathways. Together, they reflect a convergent strategy aimed at restoring neurobiological resilience rather than solely correcting neurotransmitter deficits. This integrative profile aligns with contemporary models of depression that emphasize network-level dysfunction across immune, metabolic, neuroendocrine, and synaptic systems.

From a clinical implementation standpoint, careful consideration of extract standardization, dosing, and safety is essential. While all three agents demonstrate favorable tolerability profiles, potential herb–drug interactions, particularly with anticoagulants in the case of *Ginkgo biloba* and endocrine or hepatic considerations for Ashwagandha, necessitate individualized assessment. Biomarker-informed approaches incorporating inflammatory markers, cortisol levels, cognitive status, and symptom clustering may further refine patient selection and optimize therapeutic outcomes.

Collectively, these translational insights support the incorporation of *Rhodiola rosea*, *Ginkgo biloba*, and Ashwagandha as mechanistically grounded adjuncts within evidence-based, personalized care models for depressive disorders. Their multi-target actions address biological dimensions that remain insufficiently treated by conventional monoaminergic antidepressants, highlighting their potential role in enhancing treatment responsiveness, tolerability, and long-term resilience.

## 9 Comparative and synergistic analysis of *Rhodiola rosea*, *Ginkgo biloba*, and Ashwagandha

*Rhodiola rosea*, *Ginkgo biloba*, and *Withania somnifera* (Ashwagandha) represent three botanically and pharmacologically distinct interventions that converge on core pathophysiological mechanisms of depressive disorders while maintaining clearly differentiable profiles across pharmacodynamics, pharmacokinetics, clinical efficacy, and safety. A direct comparative analysis elucidates how these agents may be positioned rationally as adjunctive or alternative interventions depending on clinical phenotype, biological vulnerability, and therapeutic goals.

### 9.1 Comparative mechanisms of action

At the level of monoaminergic modulation, *Rhodiola rosea* demonstrates the most direct enhancement of serotonergic, dopaminergic, and noradrenergic neurotransmission, mediated in part through monoamine oxidase inhibition and presynaptic modulation of neurotransmitter availability (99). This mechanism closely aligns with conventional antidepressant pharmacology, although Rhodiola exerts its effects within a broader adaptogenic framework rather than via selective receptor or transporter blockade. Ashwagandha also influences monoaminergic systems, but its effects are largely indirect, arising from normalization of hypothalamic-pituitary-adrenal (HPA) axis hyperactivity, modulation of GABAergic tone, and attenuation of stress-induced neurotransmitter imbalance. In contrast, *Ginkgo biloba* exerts comparatively modest direct monoaminergic effects; its influence on mood regulation appears secondary to improvements in neurovascular coupling, synaptic microenvironment stabilization, and antioxidant protection that preserve neurotransmitter signaling integrity rather than directly increasing monoamine levels (99, 100).

With respect to neurotrophic signaling and neuroplasticity, all three agents enhance brain-derived neurotrophic factor (BDNF)-related pathways, but through distinct biological routes. *Rhodiola rosea* upregulates BDNF expression and activates ERK/CREB signaling, thereby supporting hippocampal neurogenesis and neuronal survival. Ashwagandha promotes neurite outgrowth, synaptic protein expression, and restoration of neurotrophic deficits through combined anti-inflammatory, antioxidant, and neuroendocrine mechanisms. *Ginkgo biloba* contributes to neuroplasticity primarily by preserving mitochondrial function, reducing glucocorticoid-induced dendritic atrophy, and supporting synaptic plasticity through improved cerebral perfusion and oxidative stress reduction (8, 100).

Neuroinflammation represents a critical axis of convergence and differentiation. All three botanicals suppress pro-inflammatory cytokines such as IL-1β, IL-6, and TNF-α, yet their dominant targets differ. *Rhodiola rosea* downregulates NF-κB signaling and reduces systemic and central cytokine production. Ashwagandha robustly attenuates microglial activation and inflammatory cascades while restoring antioxidant defenses, thereby interrupting the stress-inflammation-neurotoxicity feedback loop. *Ginkgo biloba* primarily mitigates inflammation associated with oxidative stress and endothelial dysfunction, maintaining neuronal and vascular integrity rather than exerting broad immunosuppressive effects (8).

Mitochondrial protection and oxidative stress modulation further distinguish these agents. *Ginkgo biloba* demonstrates the strongest mitochondrial-stabilizing effects, including preservation of mitochondrial membrane potential, enhancement of ATP production, and reduction of cytochrome c–mediated apoptosis. *Rhodiola rosea* supports mitochondrial bioenergetics by scavenging reactive oxygen species and maintaining ATP synthesis during stress exposure. Ashwagandha enhances endogenous antioxidant enzyme systems and protects mitochondrial function indirectly through inflammation and cortisol reduction (8, 99, 100).

### 9.2 Pharmacokinetic considerations and standardization

Pharmacokinetic profiles differ substantially among the three agents and influence their clinical applicability. *Rhodiola rosea*’s antidepressant effects are largely attributed to salidroside and rosavins. Salidroside exhibits relatively rapid oral absorption, demonstrable blood–brain barrier penetration, and distribution to hippocampal and cortical regions, consistent with its central effects. However, comprehensive human pharmacokinetic data remain limited, underscoring reliance on standardized extracts such as SHR-5 with defined rosavin-to-salidroside ratios (99, 101, 102).

*Ginkgo biloba* extracts, particularly standardized formulations such as EGb 761, contain flavonoid glycosides and terpene lactones that are orally absorbed and capable of crossing the blood–brain barrier. Hepatic metabolism and elimination via urine and bile introduce interindividual variability, especially in elderly populations and in the context of polypharmacy. The necessity of restricting ginkgolic acid content highlights the critical role of standardization in ensuring safety and reproducibility (99, 101, 102).

Ashwagandha pharmacokinetics are driven by withanolides, which exhibit pleiotropic central and peripheral effects. While acute and subchronic toxicity studies suggest a wide safety margin, human pharmacokinetic data remain relatively sparse, and bioavailability may vary depending on extraction method and formulation. Advances in extraction technologies have improved withanolide recovery and batch consistency, enhancing translational reliability (99, 101, 102).

### 9.3 Comparative clinical efficacy and target populations

Clinical evidence indicates that *Rhodiola rosea* produces modest but reproducible antidepressant effects in mild to moderate depression, with dose-dependent efficacy and favorable tolerability compared to SSRIs. Its strongest clinical utility appears in stress-related depression, fatigue-dominant symptom profiles, and patient’s sensitive to antidepressant adverse effects. Combination studies with *Crocus sativus* further suggest enhanced efficacy, although placebo-controlled confirmation remains limited.

*Ginkgo biloba* demonstrates the greatest clinical benefit as an adjunctive therapy, particularly in elderly patients, individuals with cognitive impairment, and those with comorbid vascular or neurodegenerative conditions. Its capacity to accelerate antidepressant onset, improve cognition, enhance sleep architecture, and reduce biomarkers of neuronal injury positions it as a neuroprotective adjunct rather than a primary antidepressant monotherapy.

Ashwagandha exhibits broad antidepressant and anxiolytic efficacy across adult, adolescent, and geriatric populations, with particular strength in mixed anxiety–depressive states and stress-related mood disorders. Randomized controlled trials demonstrate improvements in depression, anxiety, sleep quality, quality of life, and serum serotonin levels, alongside cortisol reduction and anti-inflammatory effects. Its efficacy appears comparable to conventional antidepressants in certain settings, with superior tolerability and quality-of-life outcomes.

## 10 Limitations and future directions

Although *Rhodiola rosea*, *Ginkgo biloba*, and Ashwagandha exhibit encouraging antidepressant capabilities, various constraints presently hinder their comprehensive application in clinical settings. A principal obstacle pertains to the heterogeneity in the composition of botanical extracts, as disparities in plant provenance, extraction methodologies, and standardization protocols culminate in variable concentrations of bioactive constituents. This absence of consistency complicates the comparison of research findings, the determination of optimal dosage ranges, and the anticipation of clinical efficacy. Numerous current clinical investigations are restricted by limited sample sizes, abbreviated follow-up durations, and methodological discrepancies. Fluctuations in dosing regimens, outcome assessment criteria, and patient demographics render it arduous to derive robust conclusions pertaining to effectiveness. Furthermore, interactions between herbal supplements and pharmaceuticals remain a significant issue, particularly for individuals concurrently utilizing anticoagulants, sedatives, or thyroid-related medications. Additionally, the evidence regarding long-term safety remains inadequate, particularly concerning chronic administration and effects on vulnerable populations such as elderly individuals, pregnant women, and those with severe psychiatric comorbidities. Future investigations ought to concentrate on extensive, meticulously structured randomized clinical trials that employ standardized botanical extracts and consistent dosing methodologies. Future studies should incorporate mechanism-relevant biological markers, including serum BDNF to index neurotrophic signaling, inflammatory markers such as CRP and IL-6, measures of HPA-axis activity such as the cortisol awakening response, and neuroimaging markers (e.g., fMRI-based assessments of hippocampal connectivity), to more precisely characterize treatment response and patient stratification. Comparative investigations assessing synergistic interactions with conventional antidepressants, as well as direct comparisons among various herbal supplements, would further bolster the evidentiary foundation. Moreover, enhanced regulatory oversight pertaining to quality assurance and product labeling is imperative to guarantee reproducibility and safety. An ideal future study would employ a biomarker-stratified, randomized, triple-blind, placebo-controlled trial to evaluate the comparative efficacy and mechanistic specificity of *Rhodiola rosea*, *Ginkgo biloba*, and *Withania somnifera* as adjuncts to first-line SSRI therapy in patients with major depressive disorder. Participants with moderate depression and elevated inflammatory biomarkers, such as high-sensitivity C-reactive protein, IL-6, or TNF-α, would be enrolled to target an inflammation-positive depressive phenotype. All participants would receive a standardized SSRI regimen and be randomized to adjunctive treatment with standardized extracts of *Rhodiola rosea*, *Ginkgo biloba*, Ashwagandha, or matched placebo, with triple blinding maintained across participants, clinicians, and outcome assessors. The primary outcome would be change in depressive symptom severity over 8–12 weeks using validated clinician-rated scales, with secondary outcomes including response and remission rates, anxiety, fatigue, cognitive function, sleep quality, and quality of life. Parallel assessment of inflammatory markers, cortisol, oxidative stress indices, and neurotrophic factors such as BDNF would enable evaluation of biological target engagement and differential mechanistic effects. Safety, tolerability, and herb–drug interactions would be systematically monitored. This design would permit head-to-head and placebo-controlled comparison within a biologically defined population, advancing mechanism-guided integration of botanical adjuncts into precision treatment frameworks for depression.

Despite increasing interest in herbal interventions for mood disorders, the existing clinical literature has largely evaluated general depressive symptoms without distinguishing among specific clinical subtypes of depression. For example, a recent systematic review and meta-analysis of *Ginkgo biloba* in depressive patients reported overall efficacy on depression rating scales and related biomarkers but did not stratify outcomes by subtype such as post-stroke, postpartum, or depression comorbid with medical illness, nor was evidence on subtype-specific effects identified in the analyzed trials (103). This limitation reflects a broader gap in the field: most randomized controlled trials and systematic reviews focus on major depressive disorder or depressive symptoms in heterogeneous populations, without separate analyses for distinct presentations like vascular depression, treatment-associated depressive states, or mood disturbances in the context of specific medical conditions. As a result, the translational value of herbal therapies in these clinically relevant subgroups remains uncertain and underscores the need for future studies that explicitly evaluate efficacy and safety across different depression phenotypes.

## 11 Conclusion

*Rhodiola rosea*, *Ginkgo biloba*, and Ashwagandha encompass three noteworthy botanical agents characterized by their multifarious antidepressant efficacy. The synergistic effects of these agents on monoaminergic modulation, neurotrophic signaling, inflammatory responses, oxidative stress management, and stress-response mechanisms provide a compelling mechanistic foundation for their therapeutic significance in the treatment of depressive disorders. Empirical evidence derived from preclinical investigations reveals substantial neuroprotective, adaptogenic, and mood-enhancing attributes, whereas emerging clinical research suggests significant enhancements in mood, resilience to stress, and overall psychological well-being. Notwithstanding these promising results, the extant literature remains constrained by heterogeneous extract quality, limited clinical sample sizes, and inconsistent methodological frameworks. There exists an imperative for additional rigorously designed clinical trials and standardized formulations to substantiate efficacy, ascertain safety, and delineate optimal dosing protocols. The incorporation of these botanical supplements as adjunctive elements within a tailored, evidence-driven therapeutic paradigm may bolster treatment outcomes, mitigate adverse effects, and foster sustained mental health. Overall, these natural compounds hold significant potential to complement conventional treatments and address unmet needs in depression management, offering a holistic and biologically grounded approach to improving patient outcomes.
